# Speech Language Pathology interventions in the areas of breathing, chewing, swallowing and speaking: a scoping review

**DOI:** 10.1590/2317-1782/20232022339en

**Published:** 2023-12-18

**Authors:** Asenate Soares de Matos Pereira, Marina Gatti, Vanessa Veis Ribeiro, Karinna Veríssimo Meira Taveira, Giédre Berretin-Felix

**Affiliations:** 1 Programa de Pós-graduação em Fonoaudiologia, Faculdade de Odontologia de Bauru – FOB, Universidade de São Paulo – USP - Bauru (SP), Brasil.; 2 Universidade de Brasília – UnB - Brasília (DF), Brasil.; 3 Universidade Federal do Rio Grande do Norte – UFRN - Natal (RN), Brasil.; 4 Faculdade de Odontologia de Bauru – FOB, Universidade de São Paulo – USP - Bauru (SP), Brasil.

**Keywords:** Rehabilitation, Respiration, Mastication, Deglutition, Speech

## Abstract

There are several types of approaches that can be used to achieve therapeutic goals in disorders related to the functions of mastication, swallowing, speech, and breathing. However, the literature lacks evidence to support their use in speech-language clinical practice. The objective of this review was to map the syntheses of evidence on speech-language pathology intervention in the areas of breathing, mastication, swallowing and speech in adults and the elderly. Only studies classified by their authors as a systematic review, studies that addressed therapy for orofacial disorders in individuals over 18 years of age were included. The procedures performed included: electronic and manual search; selection of studies; data extraction; evaluation of the quality of studies and data analysis. It was possible to observe different types of interventions mainly aimed at the swallowing function, ranging from traditional therapy to the use of devices. However, due to the limitations of the studies, the data must be interpreted with caution.

## INTRODUCTION

Speech-Language Pathology is a profession whose performance encompasses several specialties, including orofacial motricity. Orofacial motricity includes assessment, diagnosis and treatment, in the different life cycles, related to sucking, breathing, mastication, musculoskeletal function related to speech and the oral phase of swallowing^(1,2)^, while oropharyngeal dysphagia comprises different phases of swallowing and includes prevention, evaluation, diagnosis, functional habilitation or rehabilitation and management of swallowing disorders^(2)^. To achieve the therapeutic goals in interventions focused on these functions, several strategies can be used, such as oral myofunctional exercises^(3)^, swallowing-related protective and facilitator maneuvers^(4)^, thermal-tactile stimulation^(5)^, and the use of devices such as neuromuscular electrostimulation^(6)^, transcranial stimulation^(7)^, among others. However, to perform a more effective and safe practice, it is important to use strategies that have scientific evidence, allowing the performance of a practice based on scientific evidence.‬ ‬‬‬‬‬‬‬‬‬‬‬‬‬

Mastication represents the initial phase of the digestive process and consists of three stages: incision, crushing and pulverizing the food, and requires good functioning and integration of the stomatognathic system muscles, teeth and temporomandibular joints. Alterations in this function may be present due to dental occlusal disorders^(8)^, temporomandibular disorder^(9)^, absence of dental elements^(10,11)^, poorly adapted prosthesis^(11)^, decrease in muscle tone and elasticity^(10)^, among others. The impairment in mastication may impact the swallowing process^(12)^, nutrition and adequate intake of nutrients^(13)^, and negatively influence the quality of life^(14)^, being essential to the intervention and performance of speech therapy in those cases.

Swallowing aims to transport the food bolus from the oral cavity to the stomach and can be divided into four phases: preparatory, oral, pharyngeal and esophageal. Swallowing has been treated by speech therapists for many years, whether in atypical or adapted swallowing in dental cases or even when it affects the individual's diet and nutrition, in neurological or oncological cases, which correspond to oropharyngeal dysphagia^(15)^. In the treatment of oropharyngeal dysphagia, speech therapists use facilitating/protective maneuvers or postural techniques, and in the clinical and instrumental diagnosis it is possible to verify which and when each of the techniques or maneuvers will be effective^(16)^. Furthermore, in the rehabilitation of oropharyngeal dysphagia, devices such as neuromuscular electrical stimulation (NMES)^(17)^ and vocal exercises^(18)^ can also be used. For oropharyngeal dysphagia, the effectiveness of therapy depends on the choice of procedures that are able to cause favorable effects on swallowing dynamics^(19)^.

Another orofacial function where speech therapy may be applied is speech. Speech production requires motor integrity - neurophysiological and neuromuscular, in addition to the components of breathing, phonation, resonance and articulation. One of the aspects that interfere with speech acquisition and production are structural alterations^(20)^, and the orofacial motricity specialist works with speech disorders resulting from neurological or musculoskeletal motor alterations. In order to diagnose the myofunctional disorders and adaptations that can cause speech alterations, it is essential to perform a morphological/structural assessment and a speech assessment through naming, repetition, automatic and informal speech^(3)^, in addition to using devices such as ultrasound, acoustic analysis of speech, comparative analysis of the photo and filming in high resolution. The speech therapy intervention is individualized and includes speech skills training and exercises, and therapeutic resources can also be used^(3)^.

In respiratory function, the nose is a highly specialized organ for humidifying, warming the air inspired and protecting the upper airways. It must be a passive conductor that captures air from the atmosphere^(21)^. Breathing can be classified as nasal, oral and mixed (nose and mouth). However, when nasal breathing is insufficient, it is replaced by oral or even mixed breathing. This insufficiency can be caused by mechanical obstacle, functional deviations or neurological dysfunction, and can present occlusal, functional and muscular alterations^(22)^. The diagnosis of oral breathing is made by the otorhinolaryngologist and the treatment can be clinical, drug-based and/or surgical, depending on the cause of the nasal obstruction^(23)^. However, an adequate treatment requires the action of a multidisciplinary team to avoid disorders that may resulting from chronic mouth breathing^(21)^, and the speech therapy intervention for the treatment of mouth breathing can include awareness and training in the breathing mode; passive maneuvers; myofunctional exercises and activities aimed at attention/perception of the breathing mode^(23)^.

There are several types of approaches (such as exercises, maneuvers, and devices) that can be used to achieve therapeutic objectives aimed at the treatment of orofacial disorders, whether in mastication, swallowing, speech or breathing. However, the literature lacks evidence to support its use in the clinical practice of speech therapy. Therefore, a scoping review is an increasingly common research method to broadly investigate the literature on a topic^(24)^, such as speech therapy interventions in the areas of breathing, mastication, swallowing and speech.

A scoping review can address broader topics, but it is still a little explored methodology in speech therapy. Therefore, it is fundamental to map the intervention studies that are being produced in speech therapy, as well as to identify research gaps in the existing literature. It is believed that such data will be able to show the current state of knowledge of interventions in orofacial functions, in addition to pointing out functions and populations in which it is necessary to expand the scope of research to provide evidence that leads to more effective and safer interventions. Therefore, the objective of this review was to map the syntheses of evidence on speech therapy intervention in the areas of breathing, mastication, swallowing and speech in adults and the elderly.

## METHODS

This scoping review was developed according to the methodology in the Joanna Briggs Institute's Reviewers' Manual^(25)^, and reported according to the Preferred Reporting Items for Systematic Reviews and Meta-Analyses extension for Scoping Reviews (PRISMA-ScR)^(26)^. The scoping review protocol has been registered, DOI 10.17605/OSF.IO/F5M4C.

### Eligibility criteria

The eligibility criteria were defined using the PCC format (Participants, Concept, Context). Eligibility for the review was as follows: Participant: studies published with population over 18 years of age (adults and the elderly); Concept: therapy; Context: breathing, mastication, swallowing and speech.

### Inclusion and exclusion criteria

Only studies classified by their authors as systematic reviews (SR), which evaluated a population of adults or elderly people, over 18 years of age, were included. Age was selected according to the age range of Health Sciences and Descriptors^(27)^, which describes adults and the elderly as people who have reached full growth or maturity. These studies must have evaluated any type of therapy for mastication, breathing, swallowing and speech functions (interventions for musculoskeletal speech disorders were considered).

Studies with a population under 18 years of age were excluded, as studies focused on childhood and adolescence need other approaches, considering the stages of development; studies in which the intervention of interest was characterized as non-speech therapy, that is, drug approaches or surgeries; descriptive studies (editorials, letters to the editor, narrative reviews, expert opinions) and primary analytical studies. There were no exclusion criteria based on ethnicity, gender, language or year of publication of the study.

### Research sources and search strategy

Eight databases of citations and abstracts were searched electronically: Cochrane, EMBASE, Latin American and Caribbean Center on Health Sciences Information (LILACS), LIVIVO, Pubmed/Medline, Scopus, SpeechByte, Web of Science. Sources of unpublished studies/Grey literature were also searched, such as Google Scholar, Open Grey, and ProQuest. Relevant keywords and vocabularies controlled in Medical Subject Headings and EMTREE were used to search each concept of interest (Appendix A) and to elaborate the final search strategies. All sources were searched as of August 9, 2021, references were managed, and duplicate studies were removed using Mendeley software (Elsevier Inc., New York, NY).

### Selection

To manage and document the review process, a Rayyan web-based application (Qatar Computing Research Institute, Qatar) was used. A pilot test of eligibility criteria was performed prior to study selection to measure inter-reviewer agreement. Good agreement was found for titles, abstracts, and full-text screening was performed only after obtaining a value > 0.7 in the Kappa Concordance Coefficient. Reviewers independently identified publications using specified and tested eligibility criteria. A third author reviewed and resolved disagreements. The same screening process was used for full-text screening. Two authors independently compiled the reference lists of review articles remaining after the full-text screening step to identify other articles of potential interest not yet retrieved. The articles identified were selected independently using previously defined eligibility criteria.

### Data extraction and storage

Two authors performed the identification and extraction of data. The data extracted included authors, year of publication, journal, impact factor; population; interventions and comparators; results; design of primary studies; PRISMA; PROSPERO; and Grading of Recommendations Assessment, Development and Evaluation (GRADE), which was then revised by two other authors. Microsoft Excel was used to compile and store the data collected.

### Quality Assessment of Systematic Reviews (SRs)

The methodological quality of the SRs included was independently assessed by two reviewers using the AMSTAR 2 checklist (a measurement tool for evaluating systematic reviews). AMSTAR 2 evaluates the methodological quality of the SRs through 16 questions that can be answered by three possible answers: “yes”, “no” or “partially yes”. The overall confidence rating (high, moderate, low, and critically low) of the studies was assessed as suggested by Shea et al.^(28)^: high: none or one non-critical weakness; moderate: more than one non-critical weakness; low: one critical failure with or without non-critical weaknesses; and critically low: more than one critical failure with or without non-critical weaknesses.

### Analysis and presentation of results

Data were analyzed descriptively and quantitatively through frequency. Data were presented in the form of figures and tables.

## Results

### Review and Selection of Primary Studies

**Figure 1:**
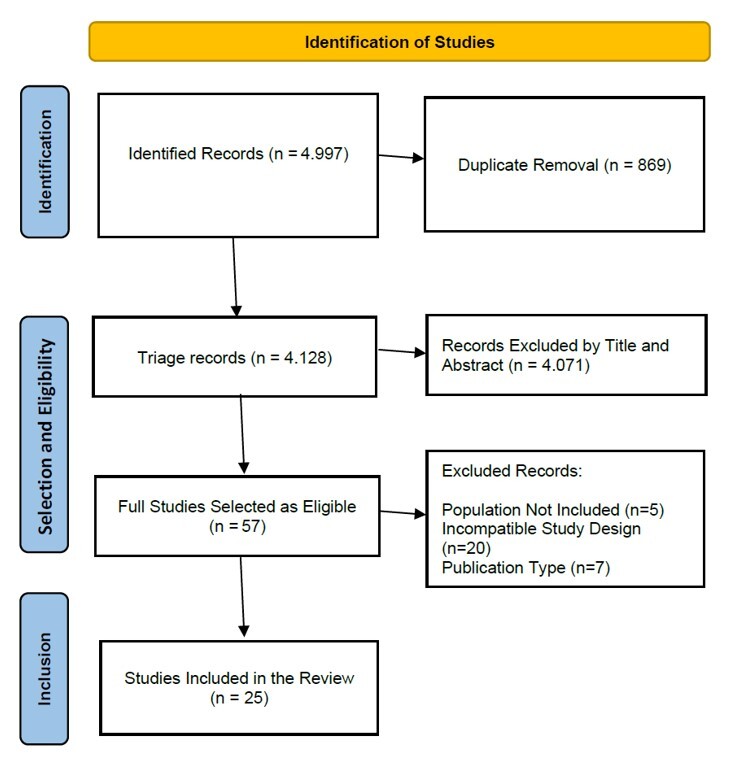
Identification of Studies

The database search strategy retrieved 4997 references, totaling 4128 after removing duplicates. During the reading phase of titles and abstracts, 57 SRs were eligible for full reading. After the reading, 32 studies were excluded, resulting in 25 SRs included for the synthesis (Figure 1).

### Characteristics of the studies

The SRs included were published between 2008 and 2021 in 21 different journals. All SRs were published in English and conducted in several countries, including Brazil^(29-32)^, South Korea^(33-35)^, Taiwan^(36,37)^, China^(38,39)^, United States^(40,41)^, Portugal^(42,43)^, Australia^(44,45)^, United Kingdom^(46,47)^, Ethiopia^(48)^, Netherlands^(49)^, Ireland^(50)^, Canada^(51)^ and Spain^(52)^.

Twenty-two studies investigated swallowing, two studies investigated speech and swallowing, one focused on breathing and none of them focused on mastication.

Participants’ ages ranged between 23 and 95 years (68% of the articles) and the majority (76%) included males and females in their sample, but in six studies this information was not found. The etiology presented by the patients in the swallowing studies was diverse, including healthy elderly, individuals after head and neck cancer treatment, patients with abnormal opening of the upper esophageal sphincter (UES), psychogenic dysphagia, neurological disorders such as brain injury, post cerebrovascular accident (CVA), traumatic brain injury (TBI), Parkinson's disease (PD) and dementia; swallowing and speech studies included patients with Parkinson's, Alzheimer's, after partial glossectomy and after stroke; and the breathing study included patients with obstructive sleep apnea.

The interventions analyzed included swallowing exercises, laryngeal and pharyngeal exercises, jaw exercises, tongue exercises, protective and facilitating swallowing maneuvers, myofunctional exercises/orofacial motor exercises, diet modification, non-oral (enteral) feeding, physical and olfactory stimulation, expiratory muscle strength training, Shaker exercise, rehabilitation exercises for speech and/or swallowing dysfunction, Chin Tuck Against Resistance (CTAR), thermal-tactile stimulation (TTS), neuromuscular electrical stimulation (NMES), electromyographic biofeedback, repetitive transcranial magnetic stimulation (rTMS), and Lee Silverman Voice Treatment (LSVT).

In the intervention groups (IG), the number of sessions ranged from one to 72 sessions, with 10 to 20 sessions being more frequent, and seven studies did not present this information^(30,40,41,43,49,51,53)^. The duration of the sessions ranged from 10 minutes to 2 hours, with 20 to 60 minutes being more frequent. In the control groups (CG), the frequency ranged from three to 30 sessions, with five sessions being more frequent. The duration of the session ranged from 10 minutes to 2 hours, with 30 minutes being more frequent, in addition to 17 studies that did not report data on frequency and duration of sessions, and seven studies that did not report whether they had CG.

The number of studies included in each SR analyzed ranged from 4 to 59, with only six performing meta-analysis. Several SRs used PRISMA as a reporting guide^(30,31,33,36,38,43-45,48,50,52)^ and five added the GRADE system^(36,38,40,48)^.

In 13 studies^(29,30,32,33,35,40,42,43,47,49,50,52,53)^ the results show the effectiveness of the analysis performed from the authors’ point of view. However, all report in their conclusion the need for further investigation, either with a more homogeneous sample, a larger number of individuals, or more rigorous methodology such as randomized controlled and blind clinical trials to validate the effectiveness of the researched aspects. They also suggest a standardization of results and analysis parameters to reduce the variability and heterogeneity of results in the short and long term. And in 7 studies^(34,37,38,44-46,51)^, the results presented were not sufficient to indicate the intervention studied, thus being inconclusive. Further information on the characteristics of the SRs can be found in Tables 1 and 2.

**Table 1:** Summary of the general descriptive characteristics of the systematic reviews included (n=25) - part I

**Author (year)**	**Population**	**Intervention Group and Control Group**	**Mentions**	**Limitations**
			**a. PRISMA**	
			**b. Protocol**	
			**c. GRADE**	
			**d. Meta-analysis**	
**Swallowing**				
Banda et al.^(36)^	Patients with HNC	**Intervention**: swallowing exercises including jaw, tongue, laryngeal and pharyngeal exercises associated with NMES and jaw devices. **Control:** usual care without exercise, while some participants underwent simulated exercises.	a. Yes; b. No; c. Yes; d. Yes	The different data formats for the same questionnaire made it difficult to assess the pooled effect and contributed to a smaller sample size in the final analysis. The use of different types of assessment tools may also have affected the overall comparisons of quality of life, making it difficult to detect any significant differences that might exist. In addition, variations in the timing and dosage of interventions made it difficult to carry out the analyses.
Park et al.^(34)^	Patients with Parkinson’s	**Intervention:** speech therapy with exercises, maneuvers, electrical stimulation and diet modification. **Control:** NI.	a. No; b. No; c. No; d. No	The data presented in the studies are still limited, and further studies are needed to investigate the effect of behavioral therapy on improving swallowing functions in patients with PD.
Alamer et al.^(48)^	Patients with post-stroke dysphagia	**Intervention**: surface neuromuscular electrical stimulation and/or combined with traditional therapy for swallowing, surface neuromuscular electrical stimulation + drug therapy; surface neuromuscular electrical stimulation + acupuncture. **Control:** traditional drug therapy and swallowing training, acupuncture, TT for dysphagia and thermal-tactile stimulation combined with exercises, inspiratory/expiratory muscle training.	a. Yes; b. No; c. Yes; d. No	In this study, only articles in English were included and due to the heterogeneity of interventions, it was not possible to perform a meta-analysis.
Albuquerque et al.^(29)^	Population with dysphagia of different etiologies.	**Intervention**: electromyographic biofeedback + traditional rehabilitation for swallowing (5 studies) electromyographic biofeedback (1 study). **Control:** traditional therapy with tongue, pharynx and larynx exercises (only 1 study had CG).	a. No; b. No; c. No; d. No	There was heterogeneity between studies and restricted use of outcome measures, and only 3 studies used instrumental swallowing tests to evaluate patients.
Andrade et al.^(30)^	Post-stroke patients with dysphagia.	**Intervention**: myofunctional orofacial exercises, laryngeal elevation, rehabilitation maneuvers and thermal stimulation (1 study); Speech therapy with postural maneuvers; for airway protection and food handling (1 study); Direct and indirect therapy using isotonic exercises, cryostimulation and bitter taste. Postural maneuvers used: cleaning and Massako (1 study); NI (2 studies). **Control**: NI.	a. Yes; b. Yes; c. No; d. No	The studies included did not analyze some variables.
Antunes et al.^(42)^	Healthy elderly, patients with abnormal UES opening, HNC, stroke.	**Intervention**: Shaker exercise. **Control:** TT (super-supraglottic maneuver, Mendelsohn maneuver and tongue base exercises) (1 study), TT (1 study), tongue pressure exercise (1 study), tongue lateralization (1 study).	a. No; b. No; c. No; d. No	The studies in this review included small sample sizes, leading to a statistical analysis with limitations. More studies are needed to evaluate the effects of the exercise in HNC and post-stroke patients, where randomized clinical trials with an appropriate sample size can provide better and more robust results.
Ashford et al.^(53)^	Secondary dysphagia to neurological disorders	**Intervention**: chin down, head rotation, Side lying, Mendelsohn and supraglottic maneuver. **Control**: NI.	a. No; b. No; c. Yes; d. No	More controlled studies on swallowing maneuvers are still needed, research using quantifiable and relevant outcome measures in specific populations.
Battel et al.^(50)^	Patients with PD.	**Intervention**: electromyographic biofeedback. **Control:** pre vs post treatment was the CG (2 studies), TT (2 studies).	a. Yes; b. Yes; c. No; d. No	Limitations included the lack of detailed reporting on how biofeedback works and the intervention applied, in addition to studies addressing long-term follow-up of the results.
Benfield et al.^(46)^	Mixed etiology (stroke, HNC, psychogenic dysphagia, TBI).	**Intervention**: biofeedback. **Control**: speech therapy without biofeedback.	a. No; b. Yes; c. No; d. Yes	Reduced number of good quality randomized clinical trials with blinding and transparent data reporting, lack of good quality observational or longitudinal studies that use pre-interventional measures as a comparator.
Carnaby and Madhavan^(41)^	population with dysphagia of different etiologies (neurological and mechanical).	**Intervention**: enteral nutrition, thicker diet modification + water on demand, behavioral treatment + NMES, Behavioral exercise + NMES, preventive behavioral exercise, maneuvers, behavioral exercise + alternative medicine, behavioral modifications and maneuvers + electrostimulation, medical and behavioral management protocols, maneuvers, postural adjustment, airway exercises. **Control:** diet modification and traditional therapy for swallowing.	a. No; b. No; c. No; d. No	Studies with small sample sizes, clinical trials that used conventional methods of simple randomization, which could interfere with the distribution of groups, only 60% of the studies included used validated outcome assessment tools, with only 26% using validated clinical and instrumental methods.
Chen et al.^(37)^	Patients with post-stroke dysphagia.	**Intervention**: neuromuscular electrical stimulation alone; swallowing therapy + neuromuscular electrical stimulation; repetitive transcranial magnetic stimulation + swallowing therapy. **Control:** swallowing therapy.	a. No; b. No; c. No; d. Yes	Heterogeneity between studies, the meta-analysis performed focused on short-term efficacy comparisons, not allowing to analyze whether neuromuscular electrical stimulation has a longer treatment effect. Also, during selection, relevant studies that were published in languages other than English may have been excluded.
Cousins et al.^(47)^	Dysphagic patients after HNC treatment.	**Intervention**: G1: Swallowing exercises only, G2: Swallowing + electrical stimulation, G3: Swallowing training + biofeedback, G4: Mandibular mobility exercises (with/without mechanical devices), G5: Devices only. **Control:** Oral motor exercises, pharyngeal swallowing exercises, use of compensatory strategies during meals, thermal/tactile stimulation, Mendelsohn's maneuver, changes in diet texture; home rehabilitation and unassisted exercise involving maximum mouth opening, lateralization and protrusion.	a. No; b. No; c. No; d. No	There are still limitations regarding evidence, requiring larger, high-quality research including patient-reported outcomes in addition to objective functional measures, allowing greater direction for future rehabilitation programs. In future studies, it is necessary to address the psychological and/or social aspects of eating and drinking.
Dionísio et al.^(43)^	Patients with post-stroke dysphagia.	**Intervention**: Transcranial Magnetic Stimulation. **Control:** NI.	a. Yes; b. Yes; c. No; d. No	The variability found between studies limited the conclusions, there was a lack of consistency in the selection of participants, protocols and outcome measures used to assess the effectiveness of the intervention. Another critical point is inter-subject variability and patient stratification.
Foley et al.^(51)^	Patients with post-stroke dysphagia.	**Intervention**: Texture-modified diets, general dysphagia therapy programs, non-oral feeding (enteral), drugs, and physical and olfactory stimulation. **Control**: NI.	a. No; b. No; c. No; d. No	Small number of studies, with heterogeneity of treatments and evaluated outcomes, preventing conclusions with definitive implications for clinical practice. More high-quality research is needed to identify effective treatments for dysphagia after stroke.
López-Liria et al.^(52)^	Patients with Parkinson's disease.	**Intervention**: oral motor exercises, airway protection maneuvers and postural compensation + NMES of the suprahyoid muscle, expiratory muscle strength training, postural techniques, swallowing exercises, traditional speech therapy, swallowing motor exercises, swallowing with effort associated with biofeedback, surface electrical stimulation, and thermal-tactile stimulation. **Control**: Oral motor exercises, airway protection maneuvers and postural compensation (CG) (1 study), expiratory muscle strength training with a device that provided little or no load (1 study). Other studies, NI.	a. Yes; b. No; c. No; d. No	Although the results of the studies included reported efficacy for the treatment of dysphagia, most of them are not of high quality. Future research with well-designed randomized controlled trials and larger populations is needed.
McCabe et al.^(40)^	Patients post HNC treatment.	**Intervention**: swallowing maneuvers (chin down, effortful swallowing, Mendelsohn, supraglottic and super-supraglottic swallowing). **Control:** NI.	a. No; b. No; c. Yes; d. No	Despite the evidence reported in the studies, larger, multicentric studies with well-defined populations and similar etiologies are needed.
Park et al.^(33)^	Post-stroke patients and healthy individuals.	**Intervention**: CTAR. **Control:** traditional treatment for dysphagia such as orofacial muscle exercises, thermal-tactile stimulation, swallowing maneuvers, several compensatory positioning procedures, or an alternative suprahyoid strengthening intervention.	a. Yes; b. No; c. No; d. No	The CTAR exercise uses equipment such as rubber balls and elastic bars, making it impossible to adjust the resistance and direction of the exercise.
Schwarz et al.^(45)^	Patients with oropharyngeal dysphagia after a neurological event or a neurodegenerative condition.	**Intervention**: thermal-tactile stimulation (TTS). **Control**: NI.	a. Yes; b. No; c. No; d. No	Limited number of articles, being of low to moderate quality. No study promoted blinding of participants or a detailed description of how raters were blinded. The search strategy and exclusion criteria used are likely to cause publication bias, as a review of the gray literature was beyond the scope of this analysis.
Speyer et al.^(49)^	Different etiologies	**Intervention**: Bolus and handling modifications, facilitating maneuvers, thermal stimulation, surface electrical stimulation, swallowing maneuvers, other interventions (Lee Silverman voice treatment, isometric tongue exercise program) and combination of interventions. **Control:** TT for swallowing.	a. No; b. No; c. No; d. No	There are several methodological issues. The control group received no therapy. Assessment of therapy outcome is based on limited groups. Some works use subjective tools to assess the effects of therapy without statistical basis or validation. It is not clear whether the data follow a random order and without any information about pre- or post-therapy status (blind classification).
Sun et al.^(38)^	Different etiologies (stroke, traumatic brain injury, HNC and PD).	**Intervention**: transcutaneous neuromuscular electrical stimulation. **Control**: simulated stimulation.	a. Yes; b. No; c. Yes; d. Yes	The studies included differed in patient characteristics, stimulation parameters, and outcome measures, contributing to heterogeneity. The meta-analysis focused on short-term efficacy immediately after the intervention, with three studies providing limited evidence of long-term efficacy and remaining controversial. This review only searched studies published in English, which may also cause bias.
Tan et al.^(39)^	Different etiologies (stroke, PD and HNC).	**Intervention**: transcutaneous neuromuscular electrical stimulation. **Control:** TT without NMES.	a. No; b. No; c. No; d. Yes	Limited design and methodological flaws, such as assessment of swallowing function being the only variable, small sample size for meta-analysis.
Yang et al.^(35)^	Patients with dysphagia after stroke	**Intervention**: transcranial direct current stimulation (tDCS) and repetitive transcranial magnetic stimulation (rTMS). **Control:** simulated stimulation.	a. No; b. No; c. No; d. Yes	The number of studies included and the sample size of each study were small. The long-term outcome after non-invasive brain stimulation could not be evaluated and its effect on dysphagia shown in these studies was only evaluated according to clinical judgment.
**Swallowing and Speech**				
Blyth et al.^(44)^	Patients after partial glossectomy.	**Intervention**: rehabilitation exercises for speech and/or swallowing dysfunction. **Control:** NI.	a. Yes; b. No; c. No; d. No	The low quality of the publications found, in terms of experimental design and level of evidence, challenges current trends in rehabilitation.
Gadenz et al.^(31)^	Patients after stroke, PD and Alzheimer's	**Intervention**: Repetitive Transcranial Magnetic Stimulation. **Control:** NI	a. Yes; b. Yes; c. No; d. No	The results were heterogeneous and limited. There was no blinding in the studies assessed.
**Breathing**				
Kayamori and Bianchini^(32)^	Patients with obstructive sleep apnea.	**Intervention:** OMT. **Control**: NI	a. No; b. No; c. No; d. No	There are few randomized controlled studies with adults, which are necessary to verify the scientific evidence that directs the parameters, both regarding the eligibility criteria for the application of OMT, and the procedures applicable in therapy.

Caption: HNC = head and neck cancer; PD = Parkinson's disease; NI = not informed; NMES = neuromuscular electrical stimulation; CTAR = Chin Tuck Against Resistance; TTS = thermal-tactile stimulation; TT = traditional therapy; tDCS = transcranial direct current stimulation; rTMS = repetitive transcranial magnetic stimulation; OMT = Orofacial Myofunctional Therapy; CG = control group; TBI = traumatic brain injury; UES = upper esophageal sphincter

**Table 2:** Summary of the General Descriptive Characteristics of the Systematic Reviews Included (n=25) - part II

**Author (year), Country, Journal, (Impact factor)**	**Objectives**	**Database Researched**	**Number, duration and frequency of sessions**	**Outcomes/Main Results**	**Main Conclusions**
**Swallowing**					
Banda et al.^(36)^, (2021) Taiwan Int J Nurs Stud (5.83)	To verify the effectiveness of swallowing exercises in improving swallowing function, performance, mouth opening, risk of aspiration/penetration and quality of life in patients with HNC.	PubMed, Medline, CINAHL, Scopus, Cochrane, Web of Science	**Intervention:** 2-5 times/day, 10 minutes-2 hours/day, 1-15 times/day or weekly. **Control:** 2-5 times/day, 10 minutes-2 hours/day, 1-15 times/day or weekly.	**- Swallowing function** (5 studies) Hedge’s g 0.33 (95%IC = 0.00–0.65) I^2^= 34.7%, p < 0.05. Swallowing exercises had little significant effect on swallowing function. **- Aspiration and penetration** (6 studies) OR 0.65 (IC 95% = 0.38–1.23, p = 0.18) I2 = 28.9%, p = 0.22). Swallowing exercises had a non-significant reduction in the risk of aspiration. **- Mouth opening** (9 studies) Hedge's g 0.60 (IC 95% = 0.21- 0.99; p < 0.003) I2= and moderate heterogeneity (Q-statistic: 24.6, I2 = 67.4%, p < 0.002). **- 6-month follow-up** (3 studies) Hedge's g 0.46 (IC 95% = 0.11–0.81, p < 0.01) and without heterogeneity (Q-statistic: 0.59, I 2 = 0%, p = 0.59). **- 1-year follow-up** (3 studies) Hedge's g 0.31 (IC 95% = −0.05; 0.66, p = 0.08) and without heterogeneity (Q-statistic: 0.69, I 2 = 0%, p = 0.71). Swallowing exercises had a significantly small effect on mouth opening in EG compared to CG immediately after the intervention and at 6-month follow-up, with no significant effect at 1-year follow-up.	Swallowing exercises were effective in improving swallowing function immediately after the intervention, and mouth opening immediately after the intervention and at the 6-month follow-up
Park et al.^(34)^, 2019 South Korea Gastroenterol Nurs (0.978)	To summarize and qualitatively analyze studies that have been published on behavioral therapies to improve swallowing functions in patients with PD.	Ovid-MEDLINE, Ovid-EMBASE, Cochrane Library and 8 Korean databases.	**Intervention:** 5-15 sessions, 20-30 min (2 studies), 5 times/week. **Control:** NI	Nine studies were included. Three studies investigated technical rehabilitation (several swallowing exercises) for the recovery of swallowing function in patients with PD, three studies investigated electrical stimulation therapy, all of which used surface electrical stimulation, and three studies investigated changes in diet and postural changes as compensatory strategies. Outcome variables were broadly divided into^(1)^ swallowing function^(2)^, swallowing-related issues, and^(2)^ assessment of quality of life and/or quality of care.	Data are insufficient to assess the effects of behavioral therapy on swallowing in PD patients, and further studies are needed.
Alamer et al.^(48)^, 2020 Ethiopia Clin Interv Aging (4.458)	To summarize the scientific evidence on the effectiveness of neuromuscular electrical stimulation on the swallowing function in post-stroke dysphagic patients.	PubMed / Medline, CINAHL, PEDro, Science Direct, Google Scholar, EMBASE and Scopus.	**Intervention:** 3-30 sessions, 10-60 min/session, 2-5 sessions/week. **Control:** 3-30 sessions, 10-60 min/session, 2-5 sessions/week.	The post-treatment effect of NMES on the swallowing function in 784 post-stroke dysphagic patients was evaluated. A total of 10 (n = 748) out of 11 studies confirmed that NMES promoted an increase in the swallowing function in post-stroke dysphagic patients compared to control groups in all outcome measures. However, one study (n = 36) indicated that NMES did not differ between EG and CG.	This review found that NMES associated with TT for swallowing could be an optional intervention to improve the swallowing function after stroke in the rehabilitation department.
Albuquerque et al.^(29)^, 2019 Brazil Eur Arch Otorhinolaryngol (2.503)	To describe the main effects of electromyographic biofeedback therapy on swallowing through a systematic review.	Scopus, Cochrane, Bireme, PubMed and via Periódicos Capes: LILACS, Medline, SciELO, Psycinfo, CINAHL.	**Intervention:**	Most studies showed positive results for the use of electromyographic biofeedback as an adjuvant therapy to improve dysphagia. In all studies, the experimental group showed significant improvement over the control group or intervention group without electromyographic biofeedback (P<0.01 and P<0.05, respectively) on all outcome measures. The combination of conventional rehabilitation with adjuvant electromyographic biofeedback was more effective in improving dysphagia than conventional rehabilitation alone.	Positive effects on the elevation of the larynx, on the improvement of swallowing functions and on the increase in the excursion and maximum elevation of the hyoid bone can be directly related to this therapy. Adjuvant therapeutic protocols with biofeedback electromyography have positive effects on the swallowing function.
			1-20 sessions, 20-60 min, 5 days to 2 weeks. **Control**:		
			20 sessions, 60 min, 15 times, 3 times/day.		
Andrade et al.^(30)^, 2017 Brazil Acta Med Port	To analyze the mean recovery time of patients with cerebrovascular accident and dysphagia submitted to speech therapy at the hospital bedside.	PubMed (including Medline), Scopus, SciELO, LILACS, OpenGrey and Google Scholar	**Intervention**: NI. **Control**: NI	A total of 5 studies out of the 5671 records screened were eligible, resulting in 176 post-stroke dysphagic individuals. The improvement in dysphagia occurred in 84.26% of the subjects and the recovery time varied between one and ninety days (mean: 22). None of the studies used randomization and blinding, management of losses, dropouts or control groups.	Speech therapy at the bedside indicates satisfactory results in a short period of time, reinforcing the importance of early diagnosis and intervention.
Antunes et al.^(42)^, 2012 Portugal Gerodontology (2.980)	Critically review the evidence on the effects of this structured intervention program and identify gaps to be filled by future studies.	PubMed, ISI Web of Knowledge, Scopus, Scielo, Lilacs	**Intervention**: 42 sessions, 1 time//day for 6 weeks, 50 min/session, 7/week. **Control**: TT 1 time/day for 6 weeks (1 study), 5 min 10 times/day (1 study), 7 sessions/week.	Comparing pre- and post-6 weeks, the exercise provided significant increase in UES opening width (p<0.05 in 3 studies; p<0.01 in 1 study), changes in thyrohyoid distance after therapy (p=0.034) (1 study), significant increase in laryngeal excursion (p<0.05 in 3 studies; p<0.01 in 1 study), significant reduction in post-swallowing aspiration to a greater degree than TT (1 study).	The data found indicate promising results of this intervention in dysphagia, although further studies are needed for a robust evaluation of the technique.
Ashford et al.^(53)^, 2009 United States J Rehabil Res Dev (1.277)	Evaluate the effectiveness of behavioral interventions for dysphagia (side lying, chin down, head rotation, effortful swallow, Mendelsohn, supraglottic swallowing, or super-supraglottic swallowing maneuvers) in relation to swallowing physiology, functional swallowing outcomes, and lung health for individuals with neurologically induced dysphagia.	PubMed; CINAHL; PsycINFO; PsycArticles; Combined Health Information Database; Health Source: Nursing, Science Citation Index; Science Direct; NeLH; REHABDATA; Social Science Citation Index; SUMSearch; TRIP Database; and Cochrane Database of Systematic Reviews, ASHA journals, National Institutes of Health Abstracts, Google Scholar and manual searches.	**Intervention**: NI. **Control**: NI.	The chin-down maneuver was effective in decreasing aspiration.	There is limited evidence of the potential effects of behavioral interventions in cases of dysphagia. Further studies are needed to evaluate its effectiveness with different populations.
				The supraglottic swallowing maneuver provided a decrease in aspiration, however its execution was difficult for patients.	
				Head rotation showed oropharyngeal efficiency and a small improvement in the opening of the cricopharyngeal anteroposterior diameter (p > 0.05). The effect size was 0.42 for oropharyngeal efficiency and 0.67 for anteroposterior cricopharyngeal opening diameter.	
				The Mendelsohn maneuver provided evolution in oral food intake.	
				The Side lying maneuver helped in the oral intake of a patient.	
Battel et al.^(50)^, 2021 Ireland Arch Phys Med Rehabil. (3.966)	To examine the effectiveness of biofeedback used in the treatment of adults with PD and dysphagia, define factors associated with biofeedback treatment outcomes, and present a theory to guide the implementation of biofeedback in future dysphagia interventions.	EMBASE, PubMed, CINAHL, Web of Science, Elsevier Scopus, ScienceDirect, AMED, The Cochrane Database of Systematic Reviews, ProQuest Dissertations and Theses A & I, Google Scholar and Grey literature.	**Intervention**: 6-18 sessions, 30-60 min, 2 weeks-3 months. **Control**: NI.	The biofeedback promoted a significant reduction in food residues, improvement in the evaluation of the DOSS scale and FOIS scale for both the intervention and control groups (1 study). Biofeedback also promoted a significant reduction in the number of coughing episodes, an improvement in voice quality (1 study), and an increase in patients' quality of life (3 studies). **2-week follow-up (1 study):** Through the timed water swallowing test, the significant reduction in the liquid swallowing rate was analyzed (p=.034). There was also a change in the EMG duration parameters of the pre-motor time (p=.003) and a significant improvement in the pre-swallowing time (p<.001).	The effectiveness of biofeedback in interventions for patients with PD and dysphagia is still uncertain, but it has shown promising results, requiring further investigation.
Benfield et al.^(46)^, 2019 United Kingdom Arch Phys Med Rehabil. (3.966)	To systematically describe and review current evidence on the effects of biofeedback-enhanced swallowing therapy in adults with dysphagia.	Cochrane Stroke Group Trials Register, MEDLINE, EMBASE, CINAHL, Conference Proceedings Citation Index-Science, and Web of Science and Grey literature.	**Intervention:** 4-72 sessions performed twice a day every fortnight, 20-60 min, with 45-60 min being most common (50%), 2week-6month treatment. **Control**: NI.	Biofeedback did not improve swallowing function (FOIS, t=2, n=51, MD=1.10; IC 95% [-1.69, 3.89], or clinical outcome (tube feeding removal, t=2, n=53, OR=3.19; IC 95% [0.16, 62.72]. The biofeedback intervention had a significant positive effect on swallowing physiology, specifically hyoid displacement (t=3, n=90, MD=0.22; IC 95% [0.04, 0.40]. There was statistically significant heterogeneity between trials in measures of swallowing function and number of tube feedings (I^2^=70%-94%) and low in physiological measures (I^2^=8%). **Accelerometry:** improvement in functional intake (FOIS) P=0.014; hyoid displacement P=0.07 (1 study). **Tongue manometry:** change in maximum isometric pressure (p=0.03), tongue swallowing pressures (P=0.014) and motor function of swallowing structures - Mann Assessment of Swallowing Capacity (p=0.04) (1 study). **EMGs:** significant improvement in duration of hyoid elevation (p=0.011) and hyoid anterior movement (p= 0.009) (2 studies); significant post-intervention changes in the biofeedback group in upper esophageal sphincter opening (p= 001), pharyngeal transit time (p=.038), and maximal hyoid displacement (p=.033) (1 study). **Videoendoscopy:** after 40 days of therapy, more patients in the biofeedback group had the tube removed and full and unrestricted oral intake (p=0.041).	Despite no evidence of improvement in functional outcomes and limited data available, therapy with biofeedback EMGs and accelerometry increased hyoid displacement.
Carnaby and Madhavan^(41)^, 2013 United States Curr Phys Med Rehabil Rep.	To further evaluate the use of rigor in recent RCT studies in dysphagia rehabilitation.	PubMed, PsychInfo, Google Scholar, EBSCO, PROQUEST Web of Science and Grey literature.	**Intervention**: 20-60 min, 2-3 times/day, 4 weeks-4 months. **Control**: 30 sec-60 min, 10 days-4 months.	Eleven (73%) RCTs reported a positive outcome of the intervention used to remedy dysphagia: improved nutritional intake, increased fluid intake, improved swallowing ability, improved quality of life, improved swallowing physiology, reduced mortality or deficiency, increased mouth opening, maintenance of chemosensory function and maintenance of swallowing muscle composition. Two studies reported negative results for their primary variable. Three studies reported no change in intervention outcome. When reviewing the design quality rating and statistical conduct of each study, five studies that reported positive results could not be substantiated due to methodological and statistical issues. Two additional studies with low methodological rigor and statistical problems identified did not report better results for their sample and remained inconclusive.	The results of the studies point to an increase in improvements with the use of RCT, although there are heterogeneous results. Further studies are needed to determine the best method of intervention.
Chen et al.^(37)^, 2016 Taiwan Clin Rehabil (3.477)	Evaluate whether swallowing treatment with NMES is superior to that without NMES, and whether NMES alone is superior to swallowing therapy.	PubMed, Scopus, Cochrane Central Register of Controlled Clinical Trials, Cochrane Systematic Reviews and ClinicalTrials.	**Intervention**: 10-20 sessions, 20-60 min, 5 sessions/week.	For the comparison “swallowing treatment with neuromuscular electrical stimulation versus swallowing treatment without neuromuscular electrical stimulation”, we found a standardized mean difference (SMD) of 1.27 (95% confidence interval (CI) = 0.51-2.02, P = 0.001) with significant heterogeneity (I^2^= 85%). The meta-analysis for the comparison of neuromuscular electrical stimulation alone and swallowing therapy showed a non-significant SMD of 0.25 (95% CI=–0.16–0.65, P = 0.23) without significant heterogeneity (I^2^= 16%).	Swallowing therapy has shown to be more effective when associated with NMES in a short-term post-stroke population. Due to the limited amount of evidence, it was not possible to indicate whether NMES alone has better results than swallowing therapy.
			**Control**: NI.		


Cousins et al.^(47)^, 2013 United Kingdom Oral Oncol. (5.337)	Identify and summarize the evidence for rehabilitation interventions aimed at alleviating eating problems after HNC treatment	Platforms OVID (Medline) and EBSCO Host (CiNAHL and PsycINFO)	**Intervention**: 10-42 sessions, 5-60 min, 3-10 times/day, 2 weeks-3 months. **Control**: NI.	Swallowing exercises (9 studies): There was a significantly greater reduction in the occurrence of post-swallowing aspiration in the Shaker group (60%) compared to the traditional group (0%) (p = 0.028; Fisher's exact test). Patients who received TT showed significant improvements in several biomechanical measures of swallowing (laryngeal movement (p=0.009) and hyoid movement (p=0.044) in swallows of 3 ml paste and anterior laryngeal movement in liquid bolus of 3 ml (p=0.026; ANOVA). The program of prophylactic swallowing exercises (involving effortful and super-supraglottic swallowing, tongue retention maneuver, tongue retraction, and Mendelsohn maneuver) provided significant differences in FOIS, which were found in favor of the intervention group at 3 and 6 months after intervention (median 3-month intervention score 7 [range 5-7] vs. median control score 5 [range 3-7] p=0.03) and median 6-month intervention score 7 [range 6-7] vs. median control score 6 [range 3-7] (p=0.009; Fisher's exact test).	Although the interventions present evidence that points to improvement in swallowing and mandibular mobility after HNC treatment, more high-quality studies are needed.
Dionísio et al.^(43)^, 2018 Portugal Cerebrovasc Dis. (2.762)	Evaluate the applicability of TMS for the rehabilitation of non-motor deficits, such as post-stroke aphasia, dysphagia and neglect.	PubMed and ISI Web of Science	**Intervention**: NI; **Control**: NI.	All articles, except one, showed qualitatively good results in improving dysphagia and were able to describe that patients recovered swallowing ability to different degrees.	The application of rTMS protocols for stroke recovery has received increasing attention in recent years, but there are still important issues that need to be investigated, the most prominent being the definition of stimulation parameters that bring the best results.
Foley et al.^(51)^, 2008 Canada Age Ageing. (10.668)	To update previous work and evaluate a wide range of therapeutic interventions intended for use in adults recovering from stroke and dysphagia.	The Cumulative Index to Nursing and Allied Health Literature (Cinahl), Medline, Embase and Cochrane Library.	**Intervention**: 1 week-1 month. **Control**: NI	Fifteen articles were retrieved evaluating a wide range of treatments that included texture-modified diets, general dysphagia therapy programs, non-oral feeding (enteral), drugs, and physical and olfactory stimulation. Among the studies, there was heterogeneity in the evaluated treatments and evaluated results, which made it impossible to use pooled analyses. Descriptively, these findings present emerging evidence that nasogastric tube feeding is not associated with an increased risk of death compared with percutaneous tube feedings; and general dysphagia therapy programs are associated with a reduced risk of pneumonia in the acute phase of stroke.	Despite recently published RCTs, few use the same treatment and outcomes, thus limiting the evidence to support the medical efficacy of common dysphagia treatments used for patients recovering from stroke.
López-Liria et al.^(52)^, 2020 Spain Int J Environ Res Public Health. (3.390)	Provide an overview of what is known about dysphagia treatments in PD, describing concise and accurate updates on advances made to date.	PubMed, Medline, Elsevier and Scopus	**Intervention**: 1-25 sessions, 20-30 min, 1-5 days/week, 1day-5week treatment. **Control**: 13–15 sessions, 20-30 min, 5 days/week, 4 week-treatment (2 studies)	The review compiles different techniques such as expiratory muscle strength training, postural techniques, oral motor exercises, video-assisted swallowing therapy, surface electrical stimulation, TTS, compensatory interventions, consistency change and electrical stimulation. Several rehabilitation therapies, such as expiratory muscle strength training or neuromuscular electrical stimulation, have been successful in swallowing and reducing the risk of asphyxia, aspiration or improving oropharyngeal function, presenting an improvement in degenerative function (coordination, speed and volume), quality of life and social relationships of individuals with PD, despite the limitations of the studies. Five articles showed improvement in the degenerative function after the application of the techniques, with a remarkable improvement in the quality of life and in the relationship of these patients with the environment. However, surface electrical stimulation did not show any positive influence on traditional speech therapy (1 study).	This review gathered several techniques and treatments used for swallowing disorders in patients with PD, such as compensation strategies, swallowing maneuvers, training of expiratory muscle strength, in addition to postural treatment, traditional physiotherapy techniques, muscle training of the tongue, pharynx, larynx and respiratory system, and surface and neuromuscular electrical stimulation. Most results obtained with the use of these techniques described in the selected articles support an improvement in degenerative function, although these results are not of high quality in most studies. Further investigations into the clinical applicability of these therapies based on well-designed randomized controlled trials are needed with larger populations for a correct estimate of effectiveness.
			Other studies, NI.		


McCabe et al.^(40)^, 2009 United States J Rehabil Res Dev. (1.277)	Answer questions about the effectiveness of interventions regarding physiology, functional swallowing outcomes for populations with structural disorders, and efficacy related to lung health	PubMed; CINAHL; PsycINFO; PsycArticles; Combined health information database; Scientific citation index; Science Direct; NeLH; REHABDATA; Social Science Citation Index; SUMSearch; TRIP Database; Cochrane Database of Systematic Reviews. Additional searches in all ASHA journals, National Institutes of Health Abstracts.	**Intervention**: NI. **Control:** NI.	**Chin-down:** helped to eliminate aspiration (1 study). **Super-supraglottic:** helped to reduce swallowing disorders (1 study), prevented aspiration in five out of nine patients (1 study), improved tongue base retraction, longer duration of tongue base contact with the posterior pharyngeal wall, and increased lingual pressure against the posterior pharyngeal wall (1 study), improved laryngeal elevation and duration of closure (2 studies), significantly reduced duration and width of upper esophageal opening (1 study). **Effortful swallow:** increased pressure of the base of the tongue with the posterior pharyngeal wall, improvement in the ability to clear thicker liquid consistencies from the pharynx. However, the maneuver may promote increased muscular effort, leading to fatigue in the fibrous tissue more quickly. **Mendelsohn maneuver:** effective in eliminating aspiration, promoting complete contact of the base of the tongue with the posterior pharyngeal wall and increasing the duration of contact (1 study), increasing the duration of consistent laryngeal elevation and improving the duration of laryngeal closure. In addition, the maneuver provided improvement in 80% of patients in oral intake by at least 1 FOIS scale score (1 study). **Supraglottic:** for the patient with composite resection of the right retromolar triangle area, the maneuver promoted little benefit in changing the base of the tongue, pharyngeal and upper esophageal opening during swallowing, and swallowing physiology (1 study).	There is currently limited evidence from six studies showing the positive effects of behavioral swallowing interventions in populations with structural disorders. Due to the range of structural deficits resulting from cancer and its treatments, further studies are needed to assess the effectiveness of the specific intervention.
Park et al.^(33)^, 2021 Korea J Oral Rehabil. (3.837)	To investigate the exercise protocols, methods and tools used in various CTAR exercise studies and summarize their findings.	Embase, Medline and Cochrane library	**Intervention:** 30-42 sessions, 30 min, 5-7 days/week, 4-8 weeks. **Control**: NI.	**CTAR vs Shaker exercise:** both promoted significant improvements in oral and pharyngeal phases (2 studies) and in PAS scores. CTAR also promoted improvement in the physiology of the oral cavity in swallowing, laryngeal elevation and epiglottic closure, reduction of vallecular residue and residue in the piriform sinuses. **CTAR exercise:** promoted significantly higher mean values and peaks of activation of the suprahyoid muscle and caused lower activation of the sternocleidomastoid muscle. **CTAR vs. traditional dysphagia treatment:** CTAR showed significantly greater improvement in PAS than traditional treatment. **CTAR vs. Shaker vs. chin flexion exercise with Theraband: s**ignificant increase in anterior tongue pressure with CTAR and with chin flexion with Theraband.	CTAR exercise more selectively activates the suprahyoid muscle and is an effective therapeutic exercise to improve swallowing function in patients with dysphagia. As it is less strenuous than the Shaker exercise, it requires less physical load and effort, allowing greater adherence.
Schwarz et al.^(45)^, 2018 Australia Int J Lang Commun Disord. (3.020)	Conduct a systematic review of the effectiveness of thermal-tactile stimulation (TTS) as a compensatory and/or rehabilitative tool.	CINAHL, Medline and SpeechBite	**Intervention**: 8-20 sessions, 30 min (reported in 1 study). **Control**: NI.	Decreased pharyngeal transit time; Median reduction in oral transit time; Decrease in the total duration of swallowing (p = 0.005); decrease in total transit time (decrease of 69%, p = 0.049 for fluids and 77%, p = 0.033 for pasty); Median reduction in pharyngeal transit time for fluids = 0.2 (85% reduction, p = 0.004), paste = 0.3 (85% reduction, p = 0.011); Median reduction in total transit time for fluids of 69%, p = 0.049 and for pasty 77%, p = 0.033); Median reduction in pharyngeal delay time for fluids of 92%, p = 0.002 and for paste reduction of 69%, p = 0.196; Better swallowing latency response using ice massage than without ice massage (p = 0.0366).	There is low-level evidence to support the use of TTS. Current best practice would be to use the TTS on a case-by-case basis, following a detailed instrumental assessment and efficacy assessment for an individual.
Speyer et al.^(49)^, 2010 Netherlands Dysphagia (3.438)	To report the effects of swallowing therapy applied by speech therapists.	PubMed and Embase	**Intervention**: NI. **Control**: NI.	A total of 59 were included and, overall, statistically significant positive effects of therapy were found. However, the number of studies was small. In addition, several methodological problems were found in many of these studies.	Comparison was hampered by the variety of diagnoses, types of therapies, and assessment techniques. Although some significant positive results studies have been published, further research based on randomized controlled trials is needed.
Sun et al.^(38)^ China Am J Phys Med Rehabil. (2.159)	To assess the effectiveness of transcutaneous neuromuscular electrical stimulation in swallowing disorders.	MEDLINE / PubMed, Embase, CENTRAL, Web of science and PEDro	**Intervention**: 10-20 sessions, 16-60 min, 2-6 times/week (mostly 5 times/week). **Control**: NI	Compared to the control groups, NMES and TT significantly improved swallowing function by a SMD of 0.62 (95% IC = 0.06 to 1.17; I2 = 89%). The SMD of the remaining eight studies was 0.92 (95% IC = 0.19 to 1.64; I2 = 90%). **Stimulation muscle groups - Studies stimulating suprahyoid muscle groups:** negative SMD value of 0.17 (95% IC = −0.42 to 0.08) without significant heterogeneity (I2 = 0%). **Studies stimulating the infrahyoid muscle groups** (SMD = 0.89; 95% IC = 0.47 to 1.30; I2 = 0%). **Studies stimulating the suprahyoid and infrahyoid muscle groups** (SMD = 1.40; 95% CI = 1.07 to 1.74; I2 = 91%). **Adverse effect:** no serious adverse effect associated with NMES was reported, only pain (2 studies), transient pain, which disappeared immediately after discontinuing NMES (1 study), and mild pain, which ceased after the adjustment of stimulation intensity (1 study).	There is no solid evidence to conclude about the effectiveness of neuromuscular electrical stimulation in swallowing disorders. Larger-scale, well-designed randomized controlled trials are needed to reach robust conclusions.
Tan et al.^(39)^, 2013 China J Oral Rehabil. (3.837)	To evaluate the overall effectiveness of transcutaneous neuromuscular electrical stimulation and TT, comparing the two treatment protocols.	PubMed/Medline, Cochrane Central Register of Controlled Trials and EMBASE MEDLINE	**Intervention:** 10-20 sessions, 30 min-1 hour, 5 times/week.	Significant improvement was seen in the NMES group compared to the TT group at SMD 0.77 (95% CI: 0.13 to 1.41, p = 0.02). The heterogeneity became small (I^2^ = 0%).	The meta-analysis showed that NMES is more effective for the treatment of adult patients with dysphagia of variable etiologies than TT. However, in patients with dysphagia after stroke, the effectiveness of NMES and TT was comparable. Considering the limitations described above, caution should be exercised when interpreting the results. We recommend that NMES is useful for the treatment of dysphagia, high quality studies with large numbers of patients are needed.
			**Control:** 13-18 sessions, 30-60 min, average Frequency of 5 sessions/week for 3 weeks	NMES was significantly superior to TT with an overall pooled score value of 0.5 (95% CI: 0.2 to 0.8, p = 0.001). It seems that the result was relatively stable. An additional sensitivity analysis was performed excluding the study in which CG patients completed treatment at home. With the overall combined score value of 0.46 (95% CI: 0.15 to 0.77, p = 0.004), there appear to be statistically significant differences between the two methods. The result was relatively stable.	
				In the subgroup analysis according to dysphagia etiology, there was no significant difference between NMES and TT in the stroke group, which had a pooled MPD value of 0.78 (95% CI: −0,22 to 1.78, p = 0·13, 4 studies, 175 patients). However, subgroup analysis of non-stroke patients, including cancer and PD patients, showed statistically significant differences between the two interventions, and the overall combined SMD value was 0.63 (95% CI: 0.24 to 1.02, = 0·002).	
Yang et al.^(35)^ South Korea Dysphagia (3.438)	To evaluate the efficacy and safety of non-invasive brain stimulation in patients with dysphagia after stroke.	Medline, EMBASE and Cochrane Library	**Intervention:** 5-10 sessions, 10-30 min, 5-7 times/week.	Statistically significant improvement in patients with dysphagia who were treated with NIBS immediately after stimulation compared to patients who underwent simulated stimulation (SMD = 1.08, 95% CI = 0.29-1.88, I^2^ = 72%). Evaluation results at 1 month after stimulation (SMD = 2.75, 95% CI = 1.47-4.04, I^2^ = 70%) and at 2 months (SMD= 3.54, 95% CI = 2.58-4.50, I^2^ = 0%) showed statistically significant improvement. Subgroup analysis based on intervention use in the rTMS group versus the simulated stimulation group (SMD = 1.61, 95% CI = 0.59-2.63, I^2^= 67%) showed significant improvement. No statistically significant difference in the tDCS group versus the simulated stimulation group (SMD = 0.54, 95% CI = -0.05-1.62, I^2^= 68%). In the subgroup analysis based on stimulation site, the contralesional site stimulation group showed statistically significant improvement compared to the sham stimulation group (SMD = 0.90, 95% CI = 0.16-1.64, I^2^ = 0%), while the ipsilesional site stimulation group showed no improvement (SMD = 1.015, 95% CI = -0.69–2.79, I^2^ = 88%). No statistically significant difference between the ipsilesional stimulation group and the contralesional stimulation group (I^2^= 0%, p = 0.87).	The results indicate that NIBS treatment for post-stroke dysphagia has a beneficial effect compared to simulated stimulation. Furthermore, NIBS reveals synergistic effects over time. In the subgroup analysis, rTMS stimulation offered beneficial effects compared to simulated stimulation. No significant differences regarding the stimulation site (ipsilesional or contralesional stimulation) were observed. No complications of NIBS were reported in this analysis. The small number of studies and the lack of long-term follow-up are the main limitations of this review. Future studies would benefit from standardization of results and stimulation parameters to decrease variability and heterogeneity of results and long-term outcomes.
			**Control**: 5-10 sessions, 10-30 min, 5-7 times/week.		
**Swallowing and Speech**					
Blyth et al.^(44)^, 2015 Australia Int J Speech Lang Pathol. (2.484)	To report speech therapy intervention in speech and swallowing after partial glossectomy.	MEDLINE, CINAHL, PubMed, EMBASE, Scopus, AMED, Web of knowledge, EBM reviews and speechBITE.	**Intervention**: 12 sessions, 30 min, 3 times/week. **Control:** NI	In all studies the therapy incorporated multiple exercises and compensations rather than a single technique. Regarding the time of intervention in the postoperative period, the start of treatment ranged from 9 days to 9 years after surgery. The studies analyzed in this article address intervention in speech, others in swallowing, 4 articles discuss intervention in dysphagia and 4 in articulation.	There are few publications on speech therapy rehabilitation after partial glossectomy, with gaps in scientific evidence.
Gadenz et al.^(31)^, 2015 Brazil Folia Phoniatr Logop. (0.849)	Systematically review randomized clinical trials that assess the effects of repetitive transcranial magnetic stimulation (rTMS) on aspects of rehabilitation related to communication and swallowing functions.	PubMed, Clinical Trials, Cochrane Library and ASHA.	**Intervention**: 5-15 sessions, 10-30 min/day, 1 time/day, 7 times/week. **Control:** 5-15 sessions, 10-30 min/day, 1 time/day, 7 times/week.	Nine studies were analyzed: 4 on aphasia, 3 on dysphagia, 1 on dysarthria in PD, and 1 on language deficits in Alzheimer's disease. All aphasia studies used low-frequency rTMS to stimulate Broca's homologous area. High-frequency rTMS was applied on the pharyngoesophageal cortex of the left and/or right hemisphere in the studies on dysphagia, and on the left dorsolateral prefrontal cortex in the studies on Parkinson's and Alzheimer's. Two studies on aphasia and all studies on dysphagia showed a significant improvement in the disorder compared to the placebo group. The other 2 studies on aphasia found benefit restricted to subgroups with severe cases or lesions in the anterior portion of the cortical area of language, respectively, while the study on Alzheimer showed specific positive effects for listening comprehension. There were no changes to vocal function in the Parkinson's study.	The benefits of the technique and its applicability in neurogenic disorders related to communication and swallowing are still uncertain. Therefore, further randomized clinical trials are needed to clarify the optimal stimulation protocol for each disorder studied and its real effects.
**Breathing**					
Kayamori and Bianchini^(32)^, 2015 Brazil Rev. CEFAC	To analyze the scientific literature regarding OMT proposals in adults with sleep-disordered breathing, as well as their effects on symptoms and physiological parameters of these disorders.	Lilacs, MEDLINE, Pubmed, Cochrane and Scielo	**Intervention:** NI	It was observed that the most relevant effects of isolated orofacial myofunctional therapy in adults include reduction in daytime sleepiness and snoring, better sleep quality, partial decrease in AHI and partial increase in minimum blood saturation. Randomized controlled and blind clinical trials are few and are important to confirm the effects of the technique based on evidence and guide therapeutic decisions considering the evaluation and diagnosis and the patient's phenotype for an accurate prognosis.	Six studies showed a decrease in the AHI, five studies showed an improvement in minimum SpO2 saturation, sleepiness scale scores and snoring. Despite the methodological differences, items that make it difficult to compare the results, the studies surveyed confirm the positive effects of OMT for patients with OSA.
			**Control**: NI		

Caption: PD = Parkinson's disease; NMES = Neuromuscular electrical stimulation; EG = experimental group; CG = control group; d = effect size-Cohen's Test; FOIS = Functional oral intake scale; DOSS = Dysphagia Outcome and Severity Scale; FEES = fiber optic endoscopic evaluation of swallowing; NI = not informed; CTAR = chin tuck against resistance; PAS = penetration-aspiration scale; rTMS = repetitive transcranial magnetic stimulation; tDCS = transcranial direct current stimulation; OSA = obstructive sleep apnea; AHI = apnea-hypopnea index; SMD = standardized mean difference; TT = traditional therapy; NIBS = Non-Invasive Neuromodulation

The SRs included reported some limitations in the primary studies, such as heterogeneity in the assessments^(29,36,43)^, in the interventions^(29,37,38,43,48,51)^ and in the population studied^(38,40,43)^; risks of selection bias in the primary studies^(45)^; limited data^(30,34,46,50,53)^; small sample^(35,39,41,42,47,52)^, limited randomized controlled^(40-42,48)^ and blind clinical trials^(31,45,46)^; methodological flaws^(37)^, use of subjective instruments in the evaluation^(35,49)^; in addition to the inclusion of studies only in the English language^(37,38)^ and with a low level of evidence^(39,44)^.

Several interventions were found in the SRs selected (Table 3). However, the most frequent were swallowing exercises/traditional therapy (60%), NMES (44%) and protective/facilitating swallowing maneuvers (40%), all related to studies on swallowing.

**Table 3:** Interventions Used

**Authors**	**Swallowing Exercises / Conventional Therapy**	**Diet Modification**	**Shaker Exercise**	**Thermal-Tactile Stimulation (TTS)**	**Neuromuscular Electrical Stimulation (NMES)**
	**Laryngeal and Pharyngeal Exercises**	**Non-Oral Feeding (Enteral)**			**Electromyographic Biofeedback**
	**Jaw Exercises**	**Thermal-Tactile, Physical, and Olfactory Stimulation**	**Rehabilitation Exercises for Speech and/or Swallowing Dysfunction**		**Repetitive Transcranial Magnetic Stimulation (rTMS)**
	**Tongue Exercises**			**Lee Silverman Voice Treatment (LSVT)**	
	**Protective/Facilitatory Swallowing Maneuvers**	**Expiratory Muscle Strength Training**	**Chin Tuck Against Resistance (CTAR)**		**Continuous Transcranial Magnetic Stimulation (cTMS)**
	**Myofunctional/Orofacial Motor Exercises**				
Banda et al.^(36)^	X	X	X	X	X
Park et al.^(34)^	X X	X			X
Alamer et al.^(48)^	X				X
Albuquerque et al.^(29)^	X				X
Andrade et al.^(30)^	X X X			X	
Antunes et al.^(42)^	X X X		X		
Ashford et al.^(53)^	X				X
Battel et al.^(50)^	X				X
Benfield et al.^(46)^	X				X
Blyth et al.^(44)^			X		
Carnaby and Madhavan^(41)^	X X	X			X
Chen et al.^(37)^	X				X X
Cousins et al.^(47)^	X X X	X		X	X X
Dionísio et al.^(43)^					X
Foley et al.^(51)^		X X X			
Gadenz et al.^(31)^					X
Kayamori e Bianchini^(32)^	X				
López-Liria et al.^(52)^	X X X	X X		X	X X
McCabe et al.^(40)^	X				
Park et al.^(33)^	X X X	X	X		
Schwarz et al.^(45)^				X	
Speyer et al.^(49)^	X X X	X		X X	X
Sun et al.^(38)^					X
Tan et al.^(39)^	X				X
Yang et al.^(35)^					X X

## INDIVIDUAL STUDY RESULTS

### Swallowing and speech

Two studies evaluated swallowing and speech. One of them analyzed patients after partial glossectomy, addressing exercises for rehabilitation of speech/swallowing dysfunction^(44)^, and the other evaluated repetitive transcranial magnetic stimulation in a population after stroke, Parkinson's or Alzheimer's^(31)^.

Data analysis was qualitative in both studies. There are still many gaps regarding speech therapy rehabilitation in the population studied^(44)^, but one of the studies^(31)^ showed the benefits of the technique applied, even though further studies are needed.

### Breathing

Only one SR^(32)^ addressed proposals for myofunctional therapy in adults with sleep-disordered breathing. The results were presented qualitatively, indicating effectiveness in reducing apnea, improving minimum saturation and improving scores on the drowsiness and snoring scale.

### Swallowing

The SRs found addressed intervention in swallowing (for cases of dysphagia and one study that included healthy elderly people) through swallowing exercises and the use of devices such as neuromuscular electrical stimulation^(36,48)^, speech therapy^(30,34,49)^, electromyographic biofeedback and/or speech therapy^(29)^, shaker exercise^(42)^, maneuvers (chin down, lateral tilt, head rotation, Mendelsohn, and supraglottic maneuver)^(53)^ (chin down, effortful swallow, Mendelsohn, supraglottic swallow, super-supraglottic swallow)^(40)^, electromyographic biofeedback^(46,50)^, neuromuscular electrical stimulation^(37)^, transcranial magnetic stimulation^(43)^, CTAR^(33)^, thermal-tactile stimulation^(45)^, transcutaneous neuromuscular electrical stimulation^(38,39)^, non-invasive brain stimulation Transcranial direct current stimulation (tDCS) and rTMS^(35)^. In four studies^(41,47,51,52)^, the authors analyzed the existing options for interventions in swallowing rehabilitation.

### Assessment of methodological quality and quality of evidence

**Figure 2:**
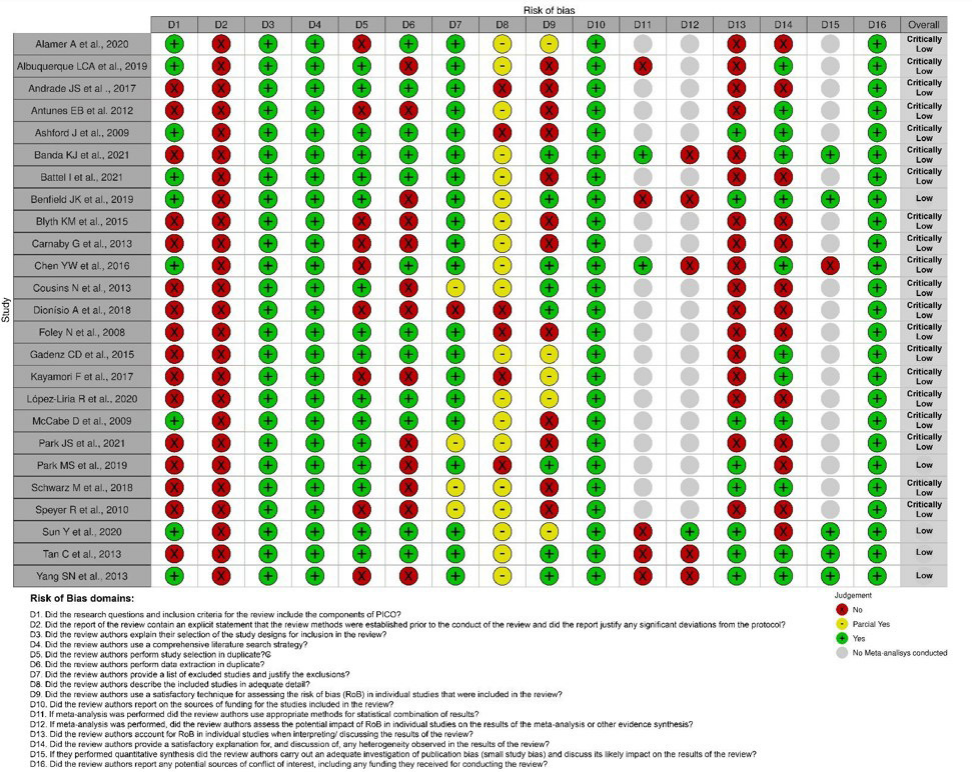
AMSTAR II: Critical Appraisal Tool for Systematic Reviews

Five SRs^(34,35,38,39,46)^ were considered to have low confidence, while the other 20 SRs^(29-33,36,37,40-45,47-53)^ were considered to have critically low confidence. None of the reviews met all the requirements of the AMSTAR guidelines (Figure 2).

All the SRs analyzed met the prerequisites of domains 3, 4, 10 and 16 of AMSTAR, which are, respectively, justifications for the selection of the study design (domain 3), search strategies (domain 4), report of the source of funding for the studies included (domain 10) and report of review authors' conflict of interest (domain 16). Only eight articles met the prerequisites for domains 9 and 13, corresponding to the technique used to assess the risk of bias in the studies included (domain 9) and risk of bias in the interpretation and discussion of results (domain 13). None of the articles complied with domain 2, which refers to reporting the protocol registration a priori.

## DISCUSSION

Bearing in mind the need in the literature for evidence of types of intervention to train orofacial functions in individuals without disorders and to rehabilitate orofacial disorders, this study performed a mapping of existing evidence through available SRs on speech therapy interventions in the areas of breathing, mastication, swallowing and speech. Twenty-five SRs that met the eligibility criteria were considered for the analysis.

The SRs included were published between 2008 and 2021, with 11 (44%) being published in the last five years^(29,33,34,36,38,43,45,46,48,50,52)^. Regarding age, most studies included patients over 50 years of age (24%), eight SRs (32%) did not report age and five (20%) reported only the mean age. This variation in the way of presenting information makes age-related inferences difficult.

Several interventions addressing swallowing were found in the literature (for cases of dysphagia and one study that included healthy elderly people); among them swallowing exercises, laryngeal and pharyngeal exercises, jaw exercises, tongue exercises, protective and facilitating swallowing maneuvers, myofunctional exercises/orofacial motor exercises, diet modification, non-oral feeding (enteral), thermal-tactile, physical and olfactory stimulation, expiratory muscle strength training, Shaker exercise, rehabilitation exercises for speech and/or swallowing dysfunction, CTAR, TTS, LSVT, in addition to treatments with the aid of devices such as NMES, electromyographic biofeedback, rTMS. Interventions addressing swallowing and speech included rrTMS and rehabilitation exercises. Interventions addressing breathing included myofunctional therapy. The most used interventions were swallowing exercises and/or traditional therapy, followed by NMES and protective and facilitating swallowing maneuvers, all related to studies on swallowing. Swallowing exercises and protective and facilitating maneuvers are proposals with proven efficacy, described over the years^(54)^. NMES has been mentioned as having an important role in several segments of dysphagia rehabilitation clinics, but authors describe the importance of specifying dysphagia etiology to prove the benefit in speech therapy practice^(17)^.

Some studies presented conclusions with positive evidence. One SR concluded that swallowing exercises were effective in improving swallowing function (immediately and after the intervention) and mouth opening (immediately, after the intervention and in the follow-up of up to six months)^(36)^. Five SRs also presented conclusions with positive scientific evidence in line with their objectives^(29,30,33,37,48)^.

There were also SRs in which the authors described the results, but reported insufficient data in their conclusions^(31,34,44)^, promising results, but lack of more robust studies^(35,41,42,46,47,50,52)^, limited evidence^(38,40,43,49,51,53)^ and low-level evidence^(45)^. These data corroborate a literature review carried out in 2007^(55)^ where the author reports the same difficulties and limitations found in studies on the effectiveness of rehabilitation in oropharyngeal dysphagia. However, it is important to highlight that the scoping study does not address the relative weight of evidence in favor of the effectiveness of the analyzed interventions, but rather a narrative or descriptive account of the available research^(56)^.

The SRs show a high level of scientific evidence, having a good study design and rigorous methodology to support the practice-based evidence (EBP), providing evidence with high reliability and lower risk of bias^(57)^. However, they are vulnerable to some types of biases, so the interpretation of the results must be done with caution. In this scoping review, the SRs showed specific limitations to secondary studies, some limitations such as heterogeneity between studies^(29,37,48,51)^, small sample sizes^(35,39,41,42)^, in addition to pointing out the need for more studies with high methodological quality^(40,42,47,51-53)^.

In domains^(2,9,15)^ of AMSTAR 2, all articles met the prerequisites. In domain 7, 20 articles (80%), and in domain 5, 16 articles (64%). On the other hand, in domain 1, only 9 articles, and in domain 6, only 12.

Among the domains considered critical^(2,3,6,8,12,14)^ by the AMSTAR 2 tool, it was observed that all articles met the prerequisites in domain 4, whereas in domain 2 none of the articles had a clear statement that the review methods were defined prior to the review. Regarding domain 7, only 4 articles (16%) partially met the prerequisites and 21 articles (84%) met all the requirements. In domains 9 and 13, 17 articles (68%) did not meet the prerequisites. In domain 15, only 6 articles (24%) performed a quantitative synthesis, in which 5 (20%) articles carried out an adequate investigation of publication bias and discussion in their results. None of the reviews met all the requirements of the AMSTAR 2 guidelines, demonstrating the need for studies with better methodological rigor.

### Implications for the research

The scoping review carried out allowed for mapping the synthesis of evidence on speech therapy interventions in the areas of swallowing, speech and breathing, in adults and the elderly. There was no SR on the mastication function. It was possible to observe different types of interventions in swallowing, from traditional therapy to the use of devices.

However, due to the limitations of the studies, the data must be interpreted with caution. Studies with high methodological quality on speech-language interventions are also needed for greater confidence inferences on evidence, which will lead to SRs with more robust studies. There are limitations in this scoping review regarding the methodological and evidence quality of the SRs included, in addition to the absence of SRs with a specific focus on safety analysis.

## CONCLUSION

The scoping review carried out allowed for mapping the synthesis of evidence on speech therapy interventions in the areas of swallowing, speech and breathing, in adults and the elderly. It was possible to observe different types of interventions in swallowing, from traditional therapy to the use of devices. However, due to the limitations of the studies, the data must be interpreted with caution. Studies with high methodological quality regarding speech-language interventions are also needed for greater confidence inferences and generalization on the evidence of efficacy and, mainly, safety.

## Appendix A Search Strategy

**Table:**

Database	Search (August 9, 2021)
Cochrane	(“therapeutics” OR “therapy” OR “Therapeutic” OR “Therapies” OR “Treatment” OR “Treatments” OR “rehabilitation” OR “intervention” OR “interventions”) in Title Abstract Keyword AND (“mastication” OR “Chewing” OR “Deglutition” OR “Deglutitions“ OR “Swallowing“ OR “Swallowings” OR “Speech” OR “Respiration” OR “Breathing”) in Title Abstract Keyword AND (“systematic review” OR “systematic reviews as topic” OR “meta-analysis” OR “meta-analysis as topic” OR “network meta-analysis” OR “network meta-analysis”) in Title Abstract Keyword
3	
Embase	('therapeutics':ab,ti OR 'therapy':ab,ti OR 'therapeutic':ab,ti OR 'therapies':ab,ti OR 'treatment':ab,ti OR 'treatments':ab,ti OR 'rehabilitation':ab,ti OR 'intervention':ab,ti OR 'interventions':ab,ti) AND ('mastication':ab,ti OR 'chewing':ab,ti OR 'deglutition':ab,ti OR deglutitions:ab,ti OR swallowing:ab,ti OR 'swallowings':ab,ti OR 'speech':ab,ti OR 'respiration':ab,ti OR 'breathing':ab,ti) AND ('systematic review':ab,ti OR 'systematic reviews as topic':ab,ti OR 'meta-analysis':ab,ti OR 'meta-analysis as topic':ab,ti OR 'network meta-analysis':ab,ti)
LILACS	(“therapeutics” OR “therapy” OR “Therapeutic” OR “Therapies” OR “Treatment” OR “Treatments” OR “rehabilitation” OR “intervention” OR “interventions” OR “Terapéutica” OR “Terapêutica” OR “Terapia” OR “Terapias” OR “Tratamento” OR “Tratamentos” OR “Speech Therapies” OR “Fonoterapia” OR “Logopedia”) AND (“mastication” OR “masticación” OR “mastigação” OR “mastication” OR “chewing” OR “Deglutição” OR “Deglutition” OR “deglutitions“ OR “swallowing“ OR “swallowings” OR “Deglución” OR “Speech” OR “Fala” OR “Habla” OR “Respiration” OR “Breathing” OR “Respiração” OR “Respiración”) AND (“systematic review” OR “Revisão Sistemática” OR “Revisión Sistemática” OR “meta-analysis” OR “meta-analysis as topic” OR “metanálise” OR “Metaanálisis” OR “network meta-analysis” OR “network meta-analysis” OR “Metanálise em Rede” OR “Metaanálisis en Red”) AND (db:(“LILACS”))
192	
LIVIVO	TI=((“therapeutics” OR “therapy” OR “Therapeutic” OR “Therapies” OR “Treatment” OR “Treatments” OR “rehabilitation” OR “intervention” OR “interventions”)) AND TI=((“mastication” OR “Chewing” OR “Deglutition” OR “Deglutitions“ OR “Swallowing“ OR “Swallowings” OR “Speech” OR “Respiration” OR “Breathing”)) AND TI=((“systematic review” OR “systematic reviews as topic” OR “meta-analysis” OR “meta-analysis as topic” OR “network meta-analysis” OR “network meta-analysis”))
3104	
PubMed/ Medline	(“therapeutics”[MesH] OR “therapeutics” OR “therapy” OR “Therapeutic” OR “Therapies” OR “Treatment” OR “Treatments” OR “rehabilitation”[MesH] OR “intervention” OR “interventions”) AND (“mastication”[MesH] OR “Chewing” OR “Deglutition”[Mesh] OR “Deglutitions“ OR “Swallowing“ OR “Swallowings” OR “Speech”[Mesh] OR “Respiration”[Mesh] OR “Breathing”) AND (“systematic review”[Publication Type] OR “systematic reviews as topic”[MesH] OR “systematic review” OR “meta-analysis” OR “meta-analysis as topic”[MesH] OR “meta-analysis” OR “network meta-analysis”[MesH] OR “network meta-analysis”)
1642	
Web of Science	(“therapeutics” OR “therapy” OR “Therapeutic” OR “Therapies” OR “Treatment” OR “Treatments” OR “rehabilitation” OR “intervention” OR “interventions”) (Topic) AND (“mastication” OR “Chewing” OR “Deglutition” OR “Deglutitions“ OR “Swallowing“ OR “Swallowings” OR “Speech” OR “Respiration” OR “Breathing”) (Topic) AND (“systematic review” OR “systematic reviews as topic” OR “meta-analysis” OR “meta-analysis as topic” OR “network meta-analysis” OR “network meta-analysis”) (Topic)
2253	
Scopus	TITLE-ABS (“therapeutics” OR “therapy” OR “Therapeutic” OR “Therapies” OR “Treatment” OR “Treatments” OR “rehabilitation” OR “intervention” OR “interventions”) AND TITLE-ABS (“mastication” OR “Chewing” OR “Deglutition” OR “Deglutitions“ OR “Swallowing“ OR “Swallowings” OR “Speech” OR “Respiration” OR “Breathing”) AND TITLE-ABS (“systematic review” OR “systematic reviews as topic” OR “meta-analysis” OR “meta-analysis as topic” OR “network meta-analysis” OR “network meta-analysis”)
ASHA Wire	(“therapeutics” OR “therapy” OR “Therapeutic” OR “Therapies” OR “Treatment” OR “Treatments” OR “rehabilitation” OR “intervention” OR “interventions”) AND (“mastication” OR “Chewing” OR “Deglutition” OR “Deglutitions“ OR “Swallowing“ OR “Swallowings” OR “Speech” OR “Respiration” OR “Breathing”) AND (“systematic review” OR “systematic reviews as topic” OR “meta-analysis” OR “meta-analysis as topic” OR “network meta-analysis” OR “network meta-analysis”)
1271	
Google Scholar	“therapeutics” AND “mastication” OR “Deglutition” OR “Speech” OR “Respiration” AND “systematic review” OR “meta-analysis”
17400	
Open Grey	“therapeutics” OR “intervention” AND “systematic review”
623	
Proquest	(“therapeutics” OR “therapy” OR “Therapeutic” OR “Therapies” OR “Treatment” OR “Treatments” OR “rehabilitation” OR “intervention” OR “interventions”) AND (“mastication” OR “Chewing” OR “Deglutition” OR “Deglutitions“ OR “Swallowing“ OR “Swallowings” OR “Speech” OR “Respiration” OR “Breathing”) AND (“systematic review” OR “systematic reviews as topic” OR “meta-analysis” OR “meta-analysis as topic” OR “network meta-analysis” OR “network meta-analysis”)
66184	
SpeechByte	“therapeutics” OR “intervention” AND “mastication” OR “Deglutition” OR “Speech” OR “Respiration” AND “systematic review” OR “meta-analysis”
	“ Treatments” AND “mastication” OR “Deglutition” OR “Speech” OR “Respiration” AND “systematic review” OR “meta-analysis”
	“Mastication” OR “Deglutition” OR “Speech” OR “Respiration” AND “systematic review” OR “meta-analysis”
	“rehabilitation” AND “mastication” OR “Deglutition” OR “Speech” OR “Respiration” AND “systematic review” OR “meta-analysis”
	“Treatments” OR “rehabilitation” OR “intervention” OR “interventions”) AND (“mastication” OR “Chewing” OR “Deglutition” OR “Deglutitions“ OR “Swallowing“ OR “Swallowings” OR “Speech” OR “Respiration” OR “Breathing”) AND (“systematic review” OR “systematic reviews as topic” OR “meta-analysis” OR “meta-analysis as topic” OR “network meta-analysis” OR “network meta-analysis”)
