# Supplementary material for: Speech Language Pathology interventions in the areas of breathing, chewing, swallowing and speaking: a scoping review
Source: Codas. 2023 Dec 18;36(2):e20220339. doi: 10.1590/2317-1782/20232022339en (PMC10750894; doi:10.1590/2317-1782/20232022339en)
Supplement: Translation

# Translation: Intervenções da Fonoaudiologia nas áreas de respiração, mastigação, deglutição e fala: uma revisão de escopo

**DOI:** 10.1590/2317-1782/20232022339pt

**Authors:** Asenate Soares de Matos Pereira, Marina Gatti, Vanessa Veis Ribeiro, Karinna Veríssimo Meira Taveira, Giédre Berretin-Felix

**Affiliations:** 1 Programa de Pós-graduação em Fonoaudiologia, Faculdade de Odontologia de Bauru – FOB, Universidade de São Paulo – USP - Bauru (SP), Brasil.; 2 Universidade de Brasília – UnB - Brasília (DF), Brasil.; 3 Universidade Federal do Rio Grande do Norte – UFRN - Natal (RN), Brasil.; 4 Faculdade de Odontologia de Bauru – FOB, Universidade de São Paulo – USP - Bauru (SP), Brasil.

**Keywords:** Reabilitação, Respiração, Mastigação, Deglutição, Fala

## Abstract

Há diversas abordagens que podem ser utilizadas para atingir os objetivos terapêuticos nos distúrbios relacionados às funções de mastigação, deglutição, fala e respiração. Entretanto, a literatura necessita de evidências que fundamentem o uso na prática clínica fonoaudiológica. O objetivo desta revisão foi mapear as sínteses de evidências sobre intervenção fonoaudiológica nas áreas de respiração, mastigação, deglutição e fala em adultos e idosos.‬ Foram incluídos apenas estudos classificados por seus autores como revisão sistemática, que abordaram terapia para disfunções orofaciais em indivíduos maiores de 18 anos. Os procedimentos realizados foram: busca eletrônica e manual; seleção dos estudos; extração dos dados; avaliação da qualidade dos estudos e análise de dados. Foi possível observar diversos tipos de intervenções principalmente voltadas à função de deglutição, abarcando desde a terapia tradicional até a utilização de dispositivos. Contudo, devido às limitações dos estudos, os dados devem ser interpretados com cautela.

## INTRODUÇÃO

A fonoaudiologia é uma profissão cuja atuação abarca diversas especialidades, dentre elas a motricidade orofacial. A motricidade orofacial inclui a avaliação, diagnóstico e tratamento nos diferentes ciclos de vida relacionada às funções de sucção, respiração, mastigação, parte musculoesquelética relacionada à fala e fase oral da deglutição^(1,2)^. Enquanto a disfagia orofaríngea compreende diferentes fases da deglutição e inclui a prevenção, avaliação, diagnóstico, habilitação ou reabilitação funcional e gerenciamento dos distúrbios da deglutição^(2)^. Para atingir os objetivos terapêuticos nas intervenções focadas nessas funções, pode-se fazer uso de diversas estratégias, como exercícios oromiofuncionais^(3)^, manobras protetoras e facilitadoras da deglutição^(4)^, estimulação tátil-térmica^(5)^, e uso de dispositivos como eletroestimulação neuromuscular^(6)^, estimulação transcraniana^(7)^, entre outras. Porém, para realizar uma prática mais eficaz e segura, é importante fazer o uso das estratégias que tenham comprovação científica, permitindo a realização de uma prática baseada em evidência científica.‬ ‬‬‬‬‬‬‬‬‬‬‬‬‬

A mastigação representa a fase inicial do processo digestório e constitui-se em três etapas: a incisão, a trituração e a pulverização do alimento. Para isso, ela requer um bom funcionamento e integração de músculos do sistema estomatognático, dentes e articulações temporomandibulares. As alterações nesta função podem estar presentes devido problemas dento-oclusais^(8)^, Disfunção Temporomandibular^(9)^, ausência de elementos dentários^(10,11)^, prótese mal adaptada^(11)^, diminuição do tônus e elasticidade muscular^(10)^, entre outros fatores. O prejuízo na mastigação pode interferir no processo de deglutição^(12)^, na nutrição e ingestão adequada de nutrientes para o indivíduo^(13)^, bem como, influenciar negativamente na qualidade de vida^(14)^, sendo assim fundamental a intervenção e atuação da fonoaudiologia nesses casos.

A deglutição tem o objetivo de transportar o bolo alimentar da cavidade oral até o estômago, ela pode ser dividida em quatro fases: preparatória, oral, faríngea e esofágica. A deglutição tem sido tratada pelos fonoaudiólogos há muitos anos, seja na deglutição atípica ou adaptada em casos odontológicos ou até mesmo na deglutição que afeta a alimentação e nutrição do indivíduo, nos casos neurológicos ou oncológicos, que correspondem as disfagias orofaríngeas^(15)^. No tratamento das disfagias orofaríngeas os fonoaudiólogos utilizam manobras facilitadoras/protetoras ou técnicas posturais, e no diagnóstico clínico e instrumental é possível verificar qual e quando cada uma das técnicas ou manobras será eficaz^(16)^. Além disso, na reabilitação da disfagia orofaríngea também pode ser feito o uso de dispositivos como eletroestimulação neuromuscular (EENM)^(17)^ e de exercícios vocais^(18)^. Para a disfagia orofaríngea, a eficácia da terapia depende da escolha de procedimentos que sejam capazes de causar efeitos favoráveis na dinâmica da deglutição^(19)^.

Outra função orofacial que a fonoaudiologia atua é a fala. A produção da fala requer integridade motora - neurofisiológica e neuromuscular, também necessita dos componentes da respiração, fonação, ressonância e articulação. Um dos aspectos que interferem na aquisição e produção da fala são as alterações estruturais^(20)^, sendo que o especialista em motricidade orofacial atua em problemas da fala decorrentes de alterações motoras neurológicas ou musculoesqueléticas. Para diagnosticar os distúrbios miofuncionais e adaptações que podem causar alterações de fala é fundamental a avaliação morfológico/estrutural e a avaliação de fala propriamente dita, através da nomeação, repetição, fala automática e informal^(3)^, além do uso de dispositivos como ultrassonografia, análise acústica da fala, análise comparativa da foto e filmagens em alta resolução. A intervenção fonoaudiológica é individualizada e abarca exercícios e treinos das habilidades de fala, podendo também serem utilizados recursos terapêuticos^(3)^.

Na função respiratória, o nariz é um órgão altamente especializado para realizar a umidificação, o aquecimento do ar que inspira e proteção das vias aéreas superiores. Ele deve ser um condutor passivo onde capta o ar da atmosfera^(21)^. A respiração pode ser classificada pelo seu modo: nasal, oral e mista (pelo nariz e pela boca). Porém, quando o modo de respiração nasal é insuficiente, passa a ser substituído pela respiração oral ou até mesmo a mista. Uma das causas desse problema é o obstáculo mecânico ou desvios funcionais e até disfunção neurológica, e pode apresentar alterações oclusal, funcional e muscular^(22)^. O diagnóstico da paciente que é respirador oral é feito pelo médico otorrinolaringologista e o tratamento pode ser clínico, medicamentoso e/ou cirúrgico, dependendo da causa da obstrução nasal^(23)^. Mas para um tratamento adequado é necessário a atuação de uma equipe multidisciplinar, evitando desordens consequentes de uma respiração bucal crônica^(21)^. E a intervenção fonoaudiológica para o tratamento da respiração oral pode incluir a conscientização e treino do modo respiratório; manobras passivas; exercícios miofuncionais e atividades dirigidas para a atenção/percepção do modo respiratório^(23)^.

Existem vários tipos de abordagens (como exercícios, manobras, dispositivos) que podem ser utilizados para atingir os objetivos terapêuticos voltados para o tratamento das disfunções orofaciais, seja na mastigação, deglutição, fala ou respiração. Entretanto, a literatura necessita de evidências que fundamentem o uso na prática clínica fonoaudiológica. Diante disso, uma revisão de escopo é um método de pesquisa cada vez mais comum para investigar amplamente a literatura sobre um tema^(24)^, como as intervenções da fonoaudiologia nas áreas de respiração, mastigação, deglutição e fala. A revisão de escopo pode abordar temas mais amplos, sendo uma metodologia pouco explorada na fonoaudiologia. Portanto, é de grande relevância um mapeamento dos estudos de intervenção que estão sendo produzidos na fonoaudiologia, bem como, uma identificação das lacunas de pesquisa na literatura existente. Acredita-se que tais dados poderão mostrar o estado da arte atual das intervenções nas funções orofaciais, além de apontar funções e populações em que se faz necessário ampliar o escopo de pesquisa para fornecer evidências que permitam a realização de intervenções mais eficazes e seguras. Por isso, o objetivo desta revisão foi mapear as sínteses de evidências sobre intervenção fonoaudiológica nas áreas de respiração, mastigação, deglutição e fala em adultos e idosos.‬ ‬ ‬‬‬

## MÉTODO

Esta revisão de escopo foi desenvolvida de acordo com a metodologia do *Joanna Briggs Institute Reviewers’ Manual* ^(25)^, e relatada de acordo com o guia para relatório de revisão de escopo *Preferred Reporting Items for Systematic reviews and Meta-Analyses extension for Scoping Reviews* (PRISMA-ScR)^(26)^. O protocolo de revisão de escopo foi registrado, DOI 10.17605/OSF.IO/F5M4C.

### Critérios de elegibilidade

Os critérios de elegibilidade foram definidos por meio do formato PCC (Participantes, Conceito, Contexto). A elegibilidade para a revisão foi a seguinte: Participante: Estudos publicados com a população maior de 18 anos (adultos e idosos); Conceito: terapia; Contexto: respiração, mastigação, deglutição e fala.

### Critérios de inclusão e exclusão

Foram incluídos apenas estudos classificados por seus autores como revisão sistemática (RS), que avaliaram uma população de indivíduos adultos ou idosos, maiores de 18 anos. A idade foi selecionada, segundo a faixa etária dos Descritores em Ciências da Saúde^(27)^, que descreve adultos e idosos como as pessoas que atingiram crescimento total ou maturidade. Esses estudos deveriam ter avaliado qualquer tipo de terapia para as funções de mastigação, respiração, deglutição e fala (foram consideradas as intervenções para alterações de fala de origem musculoesqueléticas).

Foram excluídos estudos com população abaixo de 18 anos, pois um estudo voltado para infância e adolescência precisam de outros enfoques, considerando as etapas de desenvolvimento; estudos em que a intervenção de interesse se caracterizou como terapia não fonoaudiológica, ou seja, abordagens medicamentosas ou cirúrgicas; estudos descritivos (editoriais, cartas ao editor, revisões narrativas, opiniões de especialistas) e estudos analíticos primários. Não houve critérios de exclusão com base na etnia, sexo, idioma ou ano de publicação do estudo.

### Fontes de pesquisa e estratégia de busca

Oito bases de dados de citações e resumos foram pesquisadas na busca eletrônica: Cochrane, EMBASE, Literatura Latino-Americana e do Caribe em Ciências da Saúde (LILACS), LIVIVO, Pubmed/Medline, Scopus, SpeechByte, Web of Science. Também foram pesquisadas fontes de estudos não publicados/literatura cinzenta, como o *Google Scholar, Open grey, ProQuest*. Palavras-chave relevantes e os vocabulários controlados no *Medical Subject Headings* e *EMTREE* foram usados para pesquisar cada conceito de interesse (Apêndice A) e elaborar as estratégias de busca finais. Todas as fontes foram pesquisadas em 9 de agosto de 2021, as referências foram gerenciadas e estudos duplicados foram removidos através do software Mendeley (Elsevier Inc., New York, NY).

### Seleção

Foi usado para gerenciar e documentar o processo de revisão, um aplicativo baseado na web *Rayyan (Qatar Computing Research Institute, Qatar*). Foi realizado um teste piloto sobre os critérios de elegibilidade antes da seleção do estudo para medir a concordância entre os revisores. Foi encontrada uma boa concordância para títulos, resumos e a triagem de texto completo foi feita somente após a obtenção de valor > 0.7 no Coeficiente de Concordância de Kappa. Os revisores identificaram publicações independentemente usando os critérios de elegibilidade especificados e testados. Um terceiro autor revisou e resolveu divergências. O mesmo processo de triagem foi usado para a triagem de texto completo. Dois autores compilaram de forma independente as listas de referências dos artigos de revisão restantes após a etapa de triagem de texto completo para identificar outros artigos de interesse potencial ainda não recuperados. Os artigos identificados foram selecionados de forma independente usando os critérios de elegibilidade previamente definidos.

### Extração e armazenamento dos dados

Dois autores realizaram a identificação e extração dos dados. Os dados extraídos foram: autores, ano de publicação, periódico, fator de impacto; população; intervenções e comparadores; resultados; desenho de estudos primários; PRISMA; PRÓSPERO; e Menção *Grading of Recommendations Assessment, Development and Evaluation* (GRADE), que foi então revisada por outros dois autores. O Microsoft Excel foi usado para compilação e armazenamento dos dados coletados.

### Avaliação de qualidade das revisões sistemáticas (RSs)

A qualidade metodológica das RSs incluídas foi avaliada independentemente por dois revisores usando a lista de verificação AMSTAR 2 (Uma ferramenta de medição para avaliar revisões sistemáticas). O AMSTAR 2 avalia a qualidade metodológica das RSs por meio de 16 questões que podem ser respondidas por três possibilidades de resposta: “sim”, “não” ou “parcialmente sim”. A classificação geral de confiança (alta, moderada, baixa e criticamente baixa) dos estudos foi avaliada conforme sugerido por Shea et al.^(28)^: alto: nenhuma ou uma fraqueza não crítica; moderada: mais de uma fraqueza não crítica; baixo: uma falha crítica com ou sem fraquezas não críticas; e criticamente baixo: mais de uma falha crítica com ou sem fraquezas não críticas.

### Análise e apresentação dos resultados

Os dados foram analisados de forma descritiva e quantitativa por meio de frequência. Os dados foram apresentados no formato de figuras e tabelas.

## RESULTADOS

### Revisão e seleção dos estudos primários

A estratégia de busca nas bases de dados recuperou 4997 referências, totalizando 4128 após a remoção dos duplicados. Durante a fase de leitura dos títulos e resumos, 57 RSs foram elegíveis para a leitura na íntegra. Após a leitura, 32 estudos foram excluídos, resultando em 25 RSs incluídas para a síntese (Figura 1).

**Figura 1:** Identificação dos Estudos

### Características dos estudos

As RSs incluídas foram publicadas entre 2008 e 2021 em 21 revistas diferentes. Todas as RSs foram publicadas em inglês e conduzidas em diversos países, dentre eles o Brasil^(29-32)^, Coréia do Sul^(33-35)^, Taiwan^(36,37)^, China^(38,39)^, Estados Unidos^(40,41)^, Portugal^(42,43)^, Austrália^(44,45)^, Reino Unido^(46,47)^, Etiópia^(48)^, Holanda^(49)^, Irlanda^(50)^, Canadá^(51)^ e Espanha^(52)^.

Vinte e dois estudos abordaram a área de deglutição, dois estudos a área de fala e deglutição, um a área de respiração e nenhum a área da mastigação.

A idade dos pacientes variou entre 23 e 95 anos de idade (68% dos artigos) e a maioria (76%) incluiu o gênero masculino e feminino em sua amostra, sendo que em seis estudos essa informação não foi encontrada. A etiologia apresentada pelos pacientes nos estudos de deglutição foram diversas, incluíram idosos saudáveis, indivíduos pós tratamento de câncer de cabeça e pescoço, pacientes com abertura anormal de esfíncter esofágico superior (EES), disfagia psicogênica, distúrbios neurológicos como lesão cerebral, pós acidente vascular cerebral (AVC), traumatismo cranioencefálico (TCE), doença de Parkinson (DP) e demência; os estudos de deglutição e fala incluíram pacientes com Parkinson, Alzheimer, pós glossectomia parcial e pós AVC; e o estudo de respiração incluiu pacientes com apneia obstrutiva do sono.

As intervenções analisadas abordaram exercícios de deglutição, exercícios laríngeos e faríngeos, exercícios de mandíbula, exercícios de língua, manobras protetoras e facilitadoras da deglutição, exercícios miofuncionais/motores orofaciais, modificação da dieta, alimentação não oral (enteral), estimulação física e olfatória, treinamento de força muscular expiratória, exercício com *Shaker*, exercícios para reabilitação para disfunção de fala e/ou deglutição, *Chin Tuck Against Resistance* (CTAR), estimulação tátil-térmica (ETT), EENM, *biofeedback* eletromiográfico, estimulação magnética transcraniana repetitiva (EMTr) e tratamento de voz Lee Silverman Voice Treatment (LSVT).

Nos grupos de intervenção (GI), o número de sessões variou de uma a 72 sessões, sendo mais frequente 10 a 20 sessões, além de sete estudos não descreveram essa informação^(30,40,41,43,49,51,53)^. O tempo das sessões variou de 10 minutos a 2 horas, sendo mais frequente de 20 a 60 minutos. Nos grupos controle (GC) a frequência variou de três a 30 sessões, sendo mais frequente cinco sessões; além da duração de tempo de 10 minutos a 2 horas, com 30 minutos de tempo mais frequente, além de 17 estudos não terem informado dados de frequência e tempo das sessões, e de sete estudos que não informaram se tiveram GC.

O número de estudos incluídos em cada RS analisada variou de 4 a 59 estudos, das quais apenas seis realizaram metanálise. Várias RSs utilizaram como guia de relato o PRISMA^(30,31,33,36,38,43-45,48,50,52)^ e cinco adicionaram o sistema GRADE^(36,38,40,48)^.

Em 13 estudos^(29,30,32,33,35,40,42,43,47,49,50,52,53)^ os resultados mostram efetividade sobre a análise realizada do ponto de vista dos seus autores. No entanto, todos relatam em sua conclusão a necessidade de uma investigação mais aprofundada, seja com uma amostra mais homogênea, com um maior número de indivíduos, ou com uma metodologia mais rigorosa como os ensaios clínicos randomizados controlados e cegos para validar a efetividade dos aspectos pesquisados. Também sugerem uma padronização dos resultados e dos parâmetros de análise para diminuir a variabilidade e heterogeneidade dos resultados a curto e longo prazo. E em 7 estudos^(34,37,38,44-46,51)^, os resultados apresentados não foram suficientes para indicar a intervenção estudada, sendo assim, inconclusivos. Mais informações sobre as características das RSs podem ser encontradas nas Tabelas 1 e 2.

**Tabela 1:** Resumo das características descritivas gerais das revisões sistemáticas incluídas (n=25) - parte I

**Autor**	**População**	**Grupo de Intervenção e Controle**	**Menção dos itens**	**Limitações**
			**a. PRISMA**	
			**b. Protocol**	
			**c. GRADE**	
			**d. Meta-analysis**	
**Deglutição**				
Banda et al.^(36)^	Pacientes com CCP	**Intervenção**: Exercícios de deglutição incluindo exercícios de mandíbula, língua, laríngeos e faríngeos associados a EENM e dispositivos de mandíbula. **Controle:** Receberam os cuidados usuais sem exercícios, enquanto alguns participantes dos estudos realizaram exercícios simulados.	a. Sim; b. Não; c. Sim; d. Sim	Os diferentes formatos de dados para o mesmo questionário nos estudos dificultaram a avaliação do efeito agrupado e contribuíram para um menor tamanho da amostra na análise final. O uso de diferentes tipos de instrumentos de avaliação também pode ter afetado as comparações gerais de qualidade de vida, dificultando a detecção de diferenças significativas que possam existir. Além disso, as variações no momento e na dosagem das intervenções nos estudos dificultaram a realização das análises.
Park et al.^(34)^	Pacientes com Parkinson	**Intervenção:** Terapia fonoaudiológica com exercícios, manobras, estimulação elétrica e modificação da dieta. **Controle:** NI	a. Não; b. Não; c. Não; d. Não	Os dados apresentados nos estudos ainda são limitados, sendo necessário mais estudos para investigar o efeito da terapia comportamental na melhora das funções de deglutição em pacientes com DP.
Alamer et al.^(48)^	Pacientes com disfagia pós-AVC	**Intervenção**: Estimulação elétrica neuromuscular de superfície e/ou combinadas com a terapia convencional da deglutição, estimulação elétrica neuromuscular de superfície+terapia medicamentosa; estimulação elétrica neuromuscular de superfície+acupuntura. **Controle:** Terapia medicamentosa convencional e treinamento de deglutição, acupuntura, TT para disfagia e estimulação termo-tátil com combinação de exercícios, treinamento muscular inspiratório/expiratório.	a. Sim; b. Não; c. Sim; d. Não	Neste estudo foram incluídos apenas artigos na língua inglesa e devido à heterogeneidade das intervenções não foi possível realizar uma metanálise.
Albuquerque et al.^(29)^	População com disfagia de diversas etiologias.	**Intervenção**: Biofeedback eletromiográfico + reabilitação convencional da deglutição (5 estudos) e biofeedback eletromiográfico (1 estudo). **Controle:** Terapia convencional com exercícios para língua, faringe e laringe (apenas 1 estudo teve GC).	a. Não; b. Não; c. Não; d. Não	Houve heterogeneidade entre os estudos e uso restrito de medidas de resultados, além de apenas 3 estudos terem usado exame instrumental da deglutição para avaliar os pacientes.
Andrade et al.^(30)^	Pacientes pós-AVC com disfagia.	**Intervenção**: Exercícios miofuncionais orofaciais, de elevação laríngea, manobras de reabilitação e estimulação térmica (1 estudo); Terapia da fonoaudiológica com manobras posturais; para proteção da via aérea e manipulação de alimentos (1 estudo); Terapia direta e indireta utilizando exercícios isotônicos, crioestimulação e o sabor amargo. Manobras posturais utilizadas: de limpeza e de Massako (1 estudo); NI (2 estudos). **Controle**: NI	a. Sim; b. Sim; c. Não; d. Não	Os estudos incluídos não analisaram algumas variáveis.
Antunes et al.^(42)^	Idosos saudáveis, pacientes com abertura anormal de EES, CCP, AVC.	**Intervenção**: Exercício de Shaker. **Controle:** TT (manobra super-supraglótica, manobra de Mendelsohn e exercícios de base da língua) (1 estudo), TT (1 estudo), exercício de pressão da língua (1 estudo), lateralizações da língua (1 estudo).	a. Não; b. Não; c. Não; d. Não	Os estudos nesta revisão incluíram pequenos tamanhos de amostra, ocasionando uma análise estatística com limitações. Mais estudos são necessários para avaliar os efeitos do exercício em pacientes com CCP e pós AVC, no qual os ensaios clínicos randomizados com um tamanho de amostra apropriado podem melhores resultados mais robustos.
Ashford et al.^(53)^	Disfagia secundária a distúrbios neurológicos	**Intervenção**: Queixo para baixo, rotação da cabeça, Side lying, Mendelsohn e manobra supraglótica. **Controle**: NI	a. Não; b. Não; c. Sim; d. Não	Ainda são necessários mais estudos controlados sobre manobras da deglutição, pesquisas usando medidas de resultados quantificáveis e relevantes, em populações específicas.
Battel et al.^(50)^	Pacientes com DP.	**Intervenção**: Biofeedback eletromiográfico. **Controle:** o pré vs pós-tratamento foi o GC (2 estudos), TT (2 estudos).	a. Sim; b. Sim; c. Não; d. Não	As limitações incluíram a falta de detalhes nos artigos relatando sobre o funcionamento do biofeedback, e sobre a intervenção aplicada. Além dos estudos abordarem o acompanhamento de longo prazo dos resultados.
Benfield et al.^(46)^	Etiologia mista (AVC, CCP, Disfagia psicogênica, TCE).	**Intervenção**: Biofeedback. **Controle**: terapia fonoaudiológica sem o biofeedback.	a. Não; b. Sim; c. Não; d. Sim	Número reduzido de ensaios clínicos randomizados de boa qualidade com cegamento e relatórios transparentes de dados, ausência de estudos observacionais ou longitudinais de boa qualidade que usem medidas pré-intervencionistas como um comparador.
Carnaby e Madhavan^(41)^	População com disfagia de diversas etiologias (neurológicas e mecânicas).	**Intervenção**: Nutrição enteral, modificação de dieta mais espessa + água a livre demanda, tratamento comportamental + EENM, Exercício comportamental + EENM, exercício comportamental preventivo, manobras, exercício comportamental + medicina alternativa, modificações e manobras comportamentais + eletroestimulação, protocolos de gestão médica e comportamental, manobras, ajuste postural, exercícios de vias aéreas. **Controle:** Modificação da dieta e terapia convencional da deglutição.	a. Não; b. Não; c. Não; d. Não	Foram incluídos estudos com tamanho de amostra pequenas, ensaios clínicos que usaram métodos convencionais de randomização simples podendo ter interferência na distribuição dos grupos, apenas 60% dos estudos incluídos utilizam ferramentas de avaliação de resultados validados, com apenas 26% utilizando métodos clínicos e instrumentais validados.
Chen et al.^(37)^	Pacientes com disfagia pós-AVC.	**Intervenção**: Estimulação elétrica neuromuscular sozinha; terapia da deglutição + estimulação elétrica neuromuscular; estimulação magnética transcraniana repetitiva + terapia de deglutição. **Controle:** terapia de deglutição.	a. Não; b. Não; c. Não; d. Sim	Heterogeneidade entre os estudos, a meta-análise realizada focou em comparações de eficácia de curto prazo, não permitindo analisar se a estimulação elétrica neuromuscular exerce um efeito de tratamento mais longo. Além disso, durante a seleção pode ter sido excluído estudos relevantes que foram publicados em outros idiomas que não o inglês.
Cousins et al.^(47)^	Pacientes disfágicos após tratamento de CCP.	**Intervenção**: G1: Apenas exercícios deglutição, G2: Deglutição + estimulação elétrica, G3: Treino da deglutição + biofeedback, G4: Exercícios de mobilidade mandibular (com/sem dispositivos mecânicos), G5: Apenas dispositivos. **Controle:** Exercícios motores orais, exercícios de deglutição faríngea, uso de estratégias compensatórias durante as refeições, estimulação térmica/tátil, manobra de Mendelsohn, modificações na textura da dieta; reabilitação domiciliar e exercício não assistido envolvendo a abertura de boca máxima, lateralização e protrusão.	a. Não; b. Não; c. Não; d. Não	Ainda há limitações nas evidências dos estudos, sendo necessárias pesquisas maiores e de alta qualidade, que incluem resultados relatados pelo paciente, além de medidas funcionais objetivas, permitindo um maior direcionamento para programas de reabilitação futuros. Nos estudos futuros é necessário abordar os aspectos psicológicos e/ou sociais de comer e beber.
Dionísio et al.^(43)^	Pacientes com disfagia pós-AVC.	**Intervenção**: Estimulação Magnética Transcraniana. **Controle:** NI	a. Sim; b. Sim; c. Não; d. Não	A variabilidade encontrada entre os estudos limitou as conclusões, observou-se falta de consistência quanto à seleção dos participantes, dos protocolos e das medidas de resultados que são usados para avaliar a eficácia da intervenção. Outro ponto crítico é a variabilidade intersujeitos e a estratificação dos pacientes.
Foley et al.^(51)^	Pacientes com disfagia pós-AVC.	**Intervenção**: Dietas modificadas com textura, programas de terapia geral de disfagia, alimentação não oral (enteral), medicamentos e estimulação física e olfatória. **Controle**: NI	a. Não; b. Não; c.Não; d. Não	Houve um número pequeno de estudos, com heterogeneidade dos tratamentos e desfechos avaliados, impedindo conclusões que tenham implicações definitivas para a prática clínica. São necessárias mais pesquisas de alta qualidade para identificar tratamentos eficazes para a disfagia após o AVC.
López-Liria et al.^(52)^	Pacientes com doença de Parkinson.	**Intervenção**: Exercícios motores orais, manobras de proteção de vias aéreas e compensação postural + EENM do músculo supra-hióideo, treinamento de força muscular expiratória, técnicas posturais, exercícios de deglutição, terapia fonoaudiológica tradicional, exercícios motores de deglutição, deglutição com esforço associado ao biofeedback, estimulação elétrica de superfície e estimulação termo-tátil. **Controle**: Exercícios motores orais, manobras de proteção de vias aéreas e compensação postural (GC) (1 estudo), treinamento de força muscular expiratória com um dispositivo que forneceu pouca ou nenhuma carga (1 estudo). Demais estudos NI.	a. Sim; b. Não; c. Não; d. Não	Apesar dos resultados dos estudos incluídos relatarem as eficácias para o tratamento da disfagia, sua maioria não apresenta alta qualidade. São necessárias pesquisas futuras que realizem ensaios controlados randomizados bem desenhados e com populações maiores.
McCabe et al.^(40)^	Pacientes após tratamento de CCP.	**Intervenção**: manobras da deglutição (queixo para baixo, deglutição com esforço, Mendelsohn, deglutição supraglótica e super-supraglótica). **Controle:** NI	a. Não; b. Não; c. Sim; d. Não	Apesar das evidências relatadas nos estudos, pesquisas maiores, multicêntricas e com populações bem definidas, com etiologias semelhantes são necessárias.
Park et al.^(33)^	Pacientes pós AVC e indivíduos saudáveis.	**Intervenção**: CTAR. **Controle:** Tratamento de disfagia tradicional, como exercícios musculares orofaciais, estimulação térmico-tátil, manobras de deglutição, vários procedimentos de posicionamento compensatório ou uma intervenção alternativa de fortalecimento supra-hióideo.	a. Sim; b. Não; c. Não; d. Não	O exercício CTAR usa equipamentos como bolas de borracha e barras elásticas, não possibilitando a regulagem da resistência e direção do exercício.
Schwarz et al.^(45)^	Pacientes com disfagia orofaríngea pós-evento neurológico ou quadro neurodegenerativo.	**Intervenção**: ETT. **Controle**: NI	a. Sim; b. Não; c. Não; d. Não	Número limitado de artigos, sendo eles de baixa a moderada qualidade. Em nenhum estudo houve cegamento dos participantes ou uma descrição detalhada de como os avaliadores estavam cegos. A estratégia de busca e os critérios de exclusão usados são passíveis de introduzir um viés de publicação, uma vez que uma revisão da literatura cinzenta estava além do escopo desta análise.
Speyer et al.^(49)^	Etiologias diversas	**Intervenção**: Modificações do bolo e manejo, manobras facilitadoras, estimulação térmica, estimulação elétrica de superfície, manobras de deglutição, outras intervenções (tratamento de voz Lee Silverman, programa isométrico de exercícios linguais) e combinação de intervenções. **Controle:** TT de deglutição.	a. Não; b. Não; c. Não; d. Não	Os estudos incluídos apresentam diversos problemas metodológicos. O grupo controle que não recebeu terapia. A avaliação do resultado das terapias é baseada em grupos limitados. Alguns trabalhos utilizam instrumentos subjetivos para avaliar os efeitos da terapia sem base estatística ou validação. Não estão evidentes se os dados foram pontuados em ordem aleatória e sem qualquer informação sobre o estado pré ou pós-terapia (classificação cega).
Sun et al.^(38)^	Etiologias diversas (AVC, lesão cerebral traumática, CCP e DP).	**Intervenção**: Estimulação elétrica neuromuscular transcutânea. **Controle**: Estimulação simulada	a. Sim; b. Não; c. Sim; d. Sim	Os estudos incluídos divergiram em relação às características do paciente, parâmetros de estimulação e medidas de resultados, contribuindo para a heterogeneidade. A meta-análise enfocou a eficácia de curto prazo imediatamente após a intervenção, em que apenas três estudos fornecem evidências limitadas de eficácia de longo prazo e permanecem controversos. Esta revisão buscou apenas estudos publicados em inglês, o que também pode causar viés.
Tan et al.^(39)^	Etiologias diversas (AVC, DP e CCP).	**Intervenção**: Estimulação elétrica neuromuscular transcutânea. **Controle:** TT sem EENM.	a. Não; b. Não; c. Não; d. Sim	Desenho limitado e falhas metodológicas, como a avaliação da função de deglutição sendo a única variável, amostra pequena para a meta-análise.
Yang et al.^(35)^	Pacientes com disfagia após AVC	**Intervenção**: ETCC e EMTr. **Controle:** Estimulação simulada	a. Não; b. Não; c. Não; d. Sim	O número de estudos incluídos e o tamanho da amostra de cada estudo foram pequenos. O resultado a longo prazo após a estimulação cerebral não invasiva não pôde ser avaliado e seu efeito na disfagia apresentado nestes estudos só foi avaliado de acordo com a avaliação clínica.
**Deglutição e Fala**				
Blyth et al.^(44)^	Pacientes pós glossectomia parcial.	**Intervenção**: Exercícios para reabilitação para disfunção de fala e/ou deglutição. **Controle:** NI	a. Sim; b. Não; c. Não; d. Não	A baixa qualidade das publicações encontradas, em termos de desenho experimental e nível de evidência, questiona as tendências atuais em reabilitação.
Gadenz et al.^(31)^	Pacientes pós AVC, DP e Alzheimer	**Intervenção**: Estimulação Magnética Transcraniana Repetitiva. **Controle:** NI	a. Sim; b. Sim; c. Não; d. Não	Os resultados foram heterogêneos e limitados. Não houve cegamento nos estudos analisados.
**Respiração**				
Kayamori e Bianchini^(32)^	Pacientes com apneia obstrutiva do sono.	**Intervenção:** TMO. **Controle**: NI	a. Não; b. Não; c. Não; d. Não	Existem poucos estudos randomizados controlados em adultos, sendo estes necessários para verificação de evidências científicas que direcionam os parâmetros, tanto referentes aos critérios de elegibilidade para a aplicação da TMO, quanto aos procedimentos aplicáveis em terapia.

Legenda: CCP = câncer de cabeça e pescoço; DP = doença de Parkinson; NI = não informado; EENM = estimulação elétrica neuromuscular; CTAR = Chin Tuck Against Resistance; ETT = estimulação termo tátil; TT = terapia tradicional; ETCC = estimulação transcraniana por corrente contínua; EMTr = estimulação magnética transcraniana repetitiva; TMO = Terapia Miofuncional Orofacial; GC = grupo controle; AVC = acidente vascular cerebral; TCE = traumatismo cranioencefálico; EES = esfíncter esofágico superior [[Q1: Q1]]

**Tabela 2:** Resumo das Características Descritivas Gerais das Revisões Sistemáticas Incluídas (n=25) - parte II

**Autor (ano)** **País** **Revista** **(Fator de Impacto)**	**Objetivos**	**Banco de Dados Pesquisados**	**Número, tempo e frequência das sessões**	**Desfechos/Resultados Principais**	**Principais Conclusões**
Deglutição					
Banda et al.^(36)^, (2021) Taiwan Int J Nurs Stud (5.83)	Verificar a eficácia dos exercícios de deglutição na melhora da função de deglutição, estado de desempenho, abertura da boca, risco de aspiração/penetração e qualidade de vida em pacientes com CCP.	PubMed, Medline, CINAHL, Scopus, Cochrane, Web of Science	**Intervenção:** 2-5 x por dia, dez minutos a duas horas por dia, 1 a 15 vezes por dia ou semanalmente. **Controle:** 2-5 x por dia, dez minutos a duas horas por dia, 1 a 15 vezes por dia ou semanalmente.	**- Função de deglutição** (cinco estudos) Hedge’s g 0.33 (95%IC = 0.00–0.65) I^2^= 34.7%, p < 0.05 Exercícios de deglutição tiveram um pequeno efeito significativo na função de deglutição. **- Aspiração e penetração** (seis estudos) OR 0,65 (IC 95% = 0,38–1,23, p = 0,18) I2 = 28,9%, p = 0,22). Os exercícios de deglutição tiveram redução não significativa no risco de aspiração. **- Abertura da boca** (9 estudos) Hedge's g 0,60 (IC 95% = 0,21- 0,99; p < 0,003) I2= e heterogeneidade moderada (estatística Q: 24,6, I2 = 67,4%, p < 0,002). **- Acompanhamento de 6 meses** (três estudos) Hedge's g 0,46 (IC 95% = 0,11–0,81, p < 0,01) e sem heterogeneidade (estatística Q: 0,59, I 2 = 0%, p = 0,59). **- Acompanhamento de 1 ano** (três estudos) Hedge's g 0,31 (IC 95% = −0,05; 0,66, p = 0,08) e sem heterogeneidade (estatística Q: 0,69, I 2 = 0%, p = 0,71) Os exercícios de deglutição tiveram um efeito significativamente pequeno na abertura da boca no grupo experimental em comparação com o grupo controle imediatamente após a intervenção e 6 meses de acompanhamento, sem efeito significativo no período de acompanhamento de 1 ano	Os exercícios de deglutição foram eficazes na melhora da função de deglutição imediatamente após a intervenção e na abertura da boca imediatamente após a intervenção e no acompanhamento de 6 meses
Park et al.^(34)^, 2019 Coréia do Sul Gastroenterol Nurs. (0.978)	Resumir e analisar qualitativamente os estudos que foram publicados sobre terapias comportamentais para melhorar as funções de deglutição em pacientes com DP.	Ovid-MEDLINE, Ovid-EMBASE, a Cochrane Library e 8 bancos de dados coreanos.	**Intervenção:** 5 a 15 sessões, 20 a 30 min (2 estudos), 5 vezes por semana. **Controle:** NI	9 foram estudos incluídos, três estudos examinaram a reabilitação técnica (terapias de variados exercícios de deglutição) para a recuperação da função de deglutição em pacientes com DP. Três estudos examinaram a terapia de estimulação elétrica, todos os quais usaram estimulação elétrica de superfície e outros três estudos investigaram mudanças na dieta e posturais como estratégias compensatórias. As variáveis de desfecho foram amplamente divididas em^(1)^ função da deglutição^(2)^, complicações relacionadas à deglutição, e^(2)^ avaliação da qualidade de vida e/ou qualidade do tratamento.	Os dados são insuficientes para avaliar os efeitos da terapia comportamental na deglutição de pacientes com DP, sendo necessário mais estudos.
Alamer et al.^(48)^, 2020 Etiópia Clin Interv Aging. (4.458)	Resumir as evidências científicas sobre a eficácia da estimulação elétrica neuromuscular na função de deglutição em pacientes disfágicos pós-AVC.	PubMed / Medline, CINAHL, PEDro, Science Direct, Google Scholar, EMBASE e Scopus.	**Intervenção:** 3-30 sessões, 10-60 min por sessão, 2-5 sessões por semana **Controle:** 3-30 sessões, 10-60 min, 2-5 sessões por semana.	Foi avaliado o efeito pós-tratamento da EENM na função de deglutição em pacientes com disfagia pós-AVC em 784 participantes individuais. De 11 estudos, 10 deles (n = 748) confirmaram que a EENM teve aumento da função de deglutição de pacientes com disfagia pós-AVC em comparação com os grupos de controle em todas as medidas de desfecho. No entanto, um estudo (n = 36) indicou que a EENM não teve diferenças observadas entre os grupos experimental e controle.	Esta revisão descobriu que a EENM associada à TT de deglutição pode ser uma intervenção opcional para melhorar a função de deglutição após acidente vascular cerebral no departamento de reabilitação.
Albuquerque et al.^(29)^, 2019 Brasil Eur Arch Otorhinolaryngol (2.503)	Descrever os principais efeitos da terapia de biofeedback eletromiográfico na deglutição por meio de uma revisão sistemática.	Scopus, Cochrane, Bireme, PubMed e via Periódicos Capes: LILACS, Medline, SciELO, Psycinfo, CINAHL.	**Intervenção:** 1 a 20 sessões totais, 20 a 60 min, 5 dias a 2 semanas. **Controle:** 20 sessões, 60 min, 15 vezes, três vezes ao dia.	A maioria dos estudos apresentaram resultados positivos para o uso do biofeedback eletromiográfico como terapia adjuvante na melhora da disfagia. Em todos os estudos, o grupo experimental apresentou melhora significativa em relação ao grupo controle ou grupo intervenção sem biofeedback eletromiográfico (P<0,01 e P<0,05, respectivamente) em todas as medidas de desfecho. A combinação de reabilitação convencional com biofeedback eletromiográfico adjuvante foi mais eficaz na melhora da disfagia do que a reabilitação convencional exclusiva.	Efeitos positivos na capacidade de elevação da laringe, na melhora das funções de deglutição e no aumento da excursão e elevação máxima do osso hióide podem estar diretamente relacionados a este método de terapia. Protocolos terapêuticos adjuvantes com eletromiografia de biofeedback exercem efeitos positivos na função de deglutição.
Andrade et al.^(30)^, 2017 Brasil Acta Med Port	Analisar o tempo médio de recuperação de doentes com acidente cerebrovascular encefálico e disfagia submetidos à terapia fonoaudiológica na beira do leito hospitalar.	PubMed (incluindo Medline), Scopus, SciELO, LILACS, OpenGrey e Google Scholar	**Intervenção**: NI **Controle**: NI	De 5671 registos rastreados, 5 estudos foram elegíveis. Obtiveram-se 176 indivíduos com acidente cerebrovascular encefálico e disfagia. A melhora no quadro da disfagia ocorreu em 84,26% dos sujeitos e o tempo de recuperação entre um e noventa dias (média: 22). Em nenhum estudo foi realizada a randomização e cegamento, o manejo de perdas, desistências e realizado com grupo controle.	A terapia fonoaudiológica à beira do leito indica ter resultados satisfatórios em um curto período de tempo, reforçando a importância do diagnóstico e da intervenção precoce.
Antunes et al.^(42)^, 2012 Portugal Gerodontology (2.980)	Revisar criticamente as evidências sobre os efeitos deste programa de intervenção estruturado e identificar lacunas a serem preenchidas por pesquisas futuras.	PubMed, ISI Web of Knowledge, Scopus, Scielo, Lilacs	**Intervenção**: 42 sessões, 1x ao dia durante 6 semanas, 50 min por sessão, 7x por semana. **Controle**: TT todos os dias por 6 semanas (1 estudo), 5 min 10 vezes por dia (1 estudo), 7 sessões por semana.	Em comparação pré e pós-6 semanas o exercício proporcionou aumento significativo na largura de abertura do EES (p<0,05 em 3 estudo; *p*<0,01 em 1 estudo), mudanças na distância tireo-hióidea após a terapia(p=0,034) (1 estudo), aumento significativo da excursão laríngea (p<0,05 em 3 estudo; *p*<0,01 em 1 estudo), redução significativa da aspiração pós-deglutição em maior grau do que a TT (1 estudo).	Os dados encontrados apontam resultados promissores desta intervenção em disfagia, apesar de serem necessários mais estudos para uma avaliação robusta da técnica.
Ashford et al.^(53)^, 2009 Estados Unidos J Rehabil Res Dev (1.277)	Avaliar a eficácia das intervenções comportamentais para disfagia (deitar de lado, queixo para baixo, rotação de cabeça, deglutição com esforço, Mendelsohn, supraglótica, deglutição ou manobras super-supraglóticas de deglutição) em relação a fisiologia da deglutição, resultados funcionais da deglutição e saúde pulmonar para indivíduos com disfagia induzida neurologicamente.	PubMed; CINAHL; PsycINFO; PsycArticles; Combined Health Information Database; Health Source: Nursing, Science Citation Index; Science Direct; NeLH; REHABDATA; Social Science Citation Index; SUMSearch; TRIP Database; and Cochrane Database of Systematic Reviews, ASHA journals, National Institutes of Health Abstracts, Google Scholar e pesquisas manuais.	**Intervenção**: NI **Controle**: NI	A manobra de queixo dobrado foi eficaz na diminuição da aspiração. A manobra de deglutição supraglótica também apresentou efeito de diminuição da aspiração, entretanto sua excussão foi difícil para os pacientes A rotação da cabeça apresentou eficiência orofaríngea e pequena melhora da abertura do diâmetro anteroposterior do cricofaríngeo (p > 0,05). O tamanho do efeito foi de 0,42 para eficiência orofaríngea e 0,67 para diâmetro de abertura anteroposterior do cricofaríngeo A manobra de Mendelsohn proporcionou evolução para uma ingestão oral dos alimentos. A manobra Side lying auxiliou a ingestão oral de uma paciente.	Há evidências limitadas que apresentam os potenciais efeitos das intervenções comportamentais nos casos de disfagia. São necessárias mais pesquisas para avaliar sua eficácia com diferentes populações.
Battel et al.^(50)^, 2021 Irlanda Arch Phys Med Rehabil. (3.966)	Examinar a eficácia do biofeedback usado no tratamento de adultos com DP e disfagia, definir os fatores associados aos resultados do tratamento com biofeedback e informar uma teoria para orientar a implementação do biofeedback em futuras intervenções de disfagia.	EMBASE, PubMed, CINAHL, Web of Science, Elsevier Scopus, ScienceDirect, AMED, The Cochrane Database of Systematic Reviews, ProQuest Dissertations and Theses A & I, Google Scholar e literatura cinzenta.	**Intervenção**: 6 a 18 sessões, de 30 a 60 min, 2 semanas a 3 meses. **Controle**: NI	O uso do biofeedback promoveu redução significativa nos resíduos de alimentos, melhora na avaliação da escala DOSS e na Escala FOIS tanto para o grupo de intervenção quanto para o controle (1 estudo). O biofeedback também promoveu redução significativa no número de episódios de tosse e melhora na qualidade da voz (1 estudo). Além de aumento na qualidade de vida dos pacientes (3 estudos). **Follow-up de 2 semanas (1 estudo):** Através do teste da deglutição cronometrada de água foi analisada a redução significativa da taxa de deglutição de líquidos (p=.034). Também houve mudança nos parâmetros de duração da EMGs do tempo pré-motor (p=.003) e melhora significativa do tempo pré-deglutição (p<.001).	A eficácia do biofeedback nas intervenções de pacientes com DP e disfagia ainda são incertos, porém demonstram apresentar resultados promissores, sendo necessárias maiores investigações.
Benfield et al.^(46)^, 2019 Reino Unido Arch Phys Med Rehabil. (3.966)	Descrever e revisar sistematicamente as evidências atuais sobre os efeitos da terapia de deglutição potencializada por biofeedback em adultos com disfagia.	Cochrane Stroke Group Trials Register, MEDLINE, EMBASE, CINAHL, Conference Proceedings Citation Index-Science, and Web of Science e literatura cinzenta.	**Intervenção:** 4 a 72 sessões realizadas duas vezes ao dia, quinzenalmente, 20 a 60 minutos, com 45-60 minutos sendo o mais comum (50%), tratamento com duração de 2 semanas a 6 meses. **Controle**: NI	Biofeedback não melhorou a função de deglutição (FOIS, t = 2, n = 51, MD = 1,10; IC 95% [-1,69, 3,89], ou desfecho clínico (remoção da sonda de alimentação, t =2, n=53, OR=3,19; IC 95% [0,16, 62,72]. A intervenção de biofeedback teve um efeito positivo significativo na fisiologia da deglutição, especificamente no deslocamento do hióide (t = 3, n = 90, MD = 0,22; IC 95% [0,04, 0,40] Houve heterogeneidade estatística significativa entre os ensaios em medidas de função de deglutição e número de alimentação por sonda (I^2^=70%-94%) e baixo nas medidas fisiológicas (I^2^ =8%). **- Acelerometria:** melhora da ingestão funcional (FOIS) P=0,014; o deslocamento do hióide P = 0,07 (1 estudo) **- Manometria da língua:** alteração da pressão isométrica máxima (p= 0,03), pressões de deglutição da língua (P = 0,014) e função motora das estruturas de deglutição – Avaliação de Mann da Capacidade de Deglutição (p= 0,04) (1 estudo). **- sEMG:** melhorias significativas na duração da elevação do hióide (p= 0,011) e movimento anterior do hióide (p= 0,009) (2 estudos); mudanças significativas pós-intervenção no grupo de biofeedback na abertura do esfíncter esofágico superior (p=.001), tempo de trânsito faríngeo (p=.038) e deslocamento hióide máximo (p =.033) (1 estudo). **- Videoendoscopia:** após 40 dias de terapia, mais pacientes do grupo de biofeedback apresentaram remoção da sonda e ingestão oral plena e irrestrita (p= 0,041).	Apesar de não haver evidência de melhorias nos resultados funcionais e apresentar dados limitados, a terapia com biofeedback EMGs e acelerometria aumentaram o deslocamento do hióide.
Carnaby e Madhavan^(41)^, 2013 Estados Unidos Curr Phys Med Rehabil Rep.	Para avaliar mais o uso do rigor em estudos recentes de ECR no campo da reabilitação da disfagia.	PubMed, PsychInfo, Google Scholar, EBSCO, PROQUEST Web of Science e literatura cinzenta.	**Intervenção**: 20 a 60 min, 2 a 3 vezes ao dia de 4 semanas a 4 meses. **Controle**: 30 segundos a 60 min, 10 dias a 4 meses.	Onze (73%) dos ECRs relataram um resultado positivo da intervenção usada para remediar a disfagia: melhora da ingestão nutricional, aumento da ingestão de líquidos, melhora da capacidade de engolir, melhora da qualidade de vida, melhora da fisiologia da deglutição, redução da mortalidade ou deficiência, aumento da abertura da boca, manutenção da função quimio-sensorial e manutenção da composição do músculo da deglutição. Dois estudos relataram resultados negativos para sua variável primária. Três estudos não relataram nenhuma mudança no resultado da intervenção. Ao revisar a classificação da qualidade do projeto e a conduta estatística de cada estudo, cinco estudos que relataram resultados positivos não puderam ser justificados devido a questões metodológicas e estatísticas. Dois estudos adicionais com baixo rigor metodológico e problemas estatísticos identificados não relataram melhores resultados para sua amostra e permaneceram inconclusivos.	Os dados dos estudos apontam o aumento das melhorias com a utilização da ECR, embora haja resultados heterogêneos. São necessários mais estudos para determinar o melhor método de intervenção.
Chen et al.^(37)^, 2016 Taiwan Clin Rehabil (3.477)	Avaliar se o tratamento da deglutição com EENM é superior àquele sem EENM e se a EENM isolada é superior à terapia da deglutição.	PubMed, Scopus, Cochrane Central Register of Controlled Clinical Trials, Cochrane Systematic Reviews e ClinicalTrials.	**Intervenção**: 10 a 20 sessões, 20 a 60 min, 5 sessões por semana. **Controle**: NI	Para a comparação “tratamento de deglutição com estimulação elétrica neuromuscular versus tratamento de deglutição sem estimulação elétrica neuromuscular”, encontramos uma diferença média padronizada significativa (SMD) de 1,27 (intervalo de confiança de 95% (IC) = 0,51-2,02, p = 0,001) com heterogeneidade significativa (I^2^= 85%). A meta-análise para a comparação de estimulação elétrica neuromuscular isolada e terapia de deglutição demonstrou um SMD não significativo de 0,25 (IC 95%=–0,16–0,65, p = 0,23) sem heterogeneidade significativa (I^2^= 16%).	A terapia da deglutição demonstrou ser mais eficaz quando associada a EENM, avaliando uma população pós-AVC em curto prazo. Devido ao número limitado de evidências, não foi possível indicar se a EENM isolada apresenta resultados melhores que a terapia da deglutição.
Cousins et al.^(47)^, 2013 Reino Unido Oral Oncol. (5.337)	Identificar e resumir as evidências para intervenções de reabilitação destinadas a aliviar problemas alimentares após o tratamento de CCP	Plataformas OVID (Medline) e EBSCO Host (CinAHL e PsycINFO)	**Intervenção**: 10 a 42 sessões, 5 a 60 min, 3 a 10x por dia por 2 semanas - 3 meses. **Controle**: NI	Exercícios de deglutição (9 estudos): Houve redução significativamente maior na ocorrência de aspiração pós-deglutição no grupo Shaker (60%) em relação ao grupo tradicional (0%) (p = 0,028; teste exato de Fisher). os pacientes que receberam TT demonstraram melhorias significativas em várias medidas biomecânicas da deglutição (movimento superior da laringe (p= 0,009) e movimento superior do hióide (p=0,044) em deglutições de 3 ml de pasta e movimento anterior da laringe em bolus de líquido de 3 ml (p=0,026; ANOVA). O programa de exercícios profiláticos de deglutição (envolvendo deglutição com esforço e super-supraglótica, manobra de retenção de língua, retração de língua e manobra de Mendelsohn) proporcionou diferenças significativas na FOIS, foram encontradas em favor do grupo de intervenção em 3 e 6 meses após a intervenção (escore mediano de intervenção de 3 meses 7 [intervalo 5-7] vs. escore mediano de controle 5 [intervalo 3-7] p=0,03) e escore mediano de intervenção de 6 meses 7 [intervalo 6-7] vs. escore de controle mediano 6 [intervalo 3-7] (p= 0,009; teste exato de Fisher).	Apesar das intervenções apresentarem evidências que apontam melhoras na deglutição e mobilidade mandibular após o tratamento do CCP, são necessários mais estudos de alta qualidade.
Dionísio et al.^(43)^, 2018 Portugal Cerebrovasc Dis. (2.762)	Avaliar a aplicabilidade da EMT para a reabilitação de déficits não motores, como afasia pós-AVC, disfagia e negligência.	PubMed e ISI Web of Science	**Intervenção**: NI **Controle**: NI	Todos os artigos, exceto um, demonstraram resultados qualitativamente bons na melhora da disfagia e foram capazes de descrever que os pacientes recuperaram a capacidade de deglutição em diferentes graus.	A aplicação de protocolos de EMTr para a recuperação de um acidente vascular cerebral vem recebendo cada vez mais atenção nos últimos anos, mas ainda existem questões importantes que precisam ser investigadas, sendo a mais destacada a definição dos parâmetros de estimulação que trazem os melhores resultados.
Foley et al.^(51)^, 2008 Canadá Age Ageing. (10.668)	Atualizar trabalhos anteriores e avaliar uma gama ampla de intervenções terapêuticas destinadas ao uso em adultos em recuperação de acidente vascular cerebral e disfagia.	The Cumulative Index to Nursing and Allied Health Literature (Cinahl), Medline, Embase e a Biblioteca Cochrane.	**Intervenção**: Duração de 1 semana a 1 mês. **Controle**: NI	Quinze artigos foram recuperados avaliando uma ampla gama de tratamentos que incluíam dietas modificadas com textura, programas de terapia geral de disfagia, alimentação não oral (enteral), medicamentos e estimulação física e olfatória. Entre os estudos, houve heterogeneidade dos tratamentos avaliados e os resultados avaliados que impossibilitaram o uso de análises agrupadas. Descritivamente, esses achados apresentam evidências emergentes de que a alimentação por sonda nasogástrica não está associada a um maior risco de morte em comparação com as sondas de alimentação percutâneas; e os programas gerais de terapia de disfagia estão associados a um risco reduzido de pneumonia na fase aguda do AVC.	Apesar dos ECRs recentemente publicados, poucos utilizam o mesmo tratamento e resultados, limitando assim as evidências para apoiar a eficácia médica dos tratamentos comuns de disfagia usados para pacientes em recuperação de acidente vascular cerebral.
López-Liria et al.^(52)^, 2020 Espanha Int J Environ Res Public Health. (3.390)	Fornecer uma visão geral do que se sabe sobre os tratamentos da disfagia na DP, descrevendo atualizações concisas e precisas sobre os avanços alcançados até o momento.	PubMed, Medline, Elsevier e Scopus	**Intervenção**: 1 a 25 sessões, 20 a 30 min, 1 a 5 dias por semana e tratamento com duração de 1 dia a 5 semanas. **Controle**: 13–15 sessões, 20 a 30 min, 5 dias por semana, tratamento com duração de 4 semanas (2 estudos) Demais estudos NI.	A revisão compila diferentes técnicas como treinamento de força muscular expiratória, técnicas posturais, exercícios motores orais, terapia videoassistida da deglutição, estimulação elétrica de superfície, ETT, intervenções compensatórias, alteração de consistência e estimulação elétrica. Várias terapias de reabilitação como o treinamento de força muscular expiratória ou a estimulação elétrica neuromuscular têm sido bem-sucedidas na deglutição e na redução do risco de asfixia, aspiração ou melhora da função orofaríngea. Sendo analisada uma melhora na função degenerativa (coordenação, velocidade e volume), qualidade de vida e relações sociais de pessoas com DP, apesar dos estudos apresentarem limitações. 5 artigos observaram melhora da função degenerativa após a aplicação das técnicas empregadas e, assim, foi notável a melhora na qualidade de vida e na relação desses pacientes com o meio ambiente. No entanto, a estimulação elétrica de superfície não mostrou qualquer influência positiva na terapia fonoaudiológica tradicional (1 estudo).	Esta revisão reuniu diversas técnicas e tratamentos utilizados para problemas de deglutição em pacientes com DP, como estratégias de compensação, manobras de deglutição, treinamento de força muscular expiratória, juntamente com tratamento postural, técnicas tradicionais de fisioterapia, treinamento muscular de língua, faringe, laringe e aparelho respiratório e estimulação elétrica de superfície e neuromuscular. A maioria dos resultados obtidos com a aplicação dessas técnicas descritas nos artigos selecionados suportam uma melhora na função degenerativa, embora esses resultados não sejam de alta qualidade na maioria dos estudos. Outras investigações sobre a aplicabilidade clínica dessas terapias baseadas em ensaios controlados randomizados bem desenhados são necessárias com populações maiores de pacientes. Isso dará uma estimativa correta da eficácia.
McCabe et al.^(40)^, 2009 Estados Unidos J Rehabil Res Dev. (1.277)	Responder às questões sobre a eficácia das intervenções, quanto a fisiologia, resultados funcionais da deglutição para populações com um distúrbio estrutural e eficácia relacionada a saúde pulmonar	PubMed; CINAHL; PsycINFO; PsycArticles; Banco de dados de informações de saúde combinados; Índice de citações científicas; Science Direct; NeLH; REHABDATA; Índice de Citação de Ciências Sociais; SUMSearch; Banco de dados TRIP; Cochrane Database of Systematic Reviews. Pesquisas adicionais em todas as revistas ASHA, National Institutes of Health Abstracts.	**Intervenção**: NI **Controle:** NI	**Queixo para baixo:** auxiliou a eliminar a aspiração (1 estudo) **Super-supraglótica:** auxiliou para a redução dos distúrbios da deglutição (1 estudo), preveniu a aspiração em cinco de nove pacientes (1 estudo), promoveu melhora da retração da base da língua, durações mais longas de contato da base da língua com a parede posterior da faringe e aumentos na pressão lingual contra a parede posterior da faringe (1 estudo), melhora da elevação laríngea e duração de seu fechamento (2 estudos), redução significativa da duração e a largura da abertura do esôfago superior (1 estudo). **Deglutição com esforço:** produziu aumento da pressão do contato da base da língua com a parede posterior da faringe, melhora na capacidade de limpar consistências líquidas mais espessas da faringe. Porém, a manobra pode promover aumento do esforço muscular necessário levando a fadiga mais rapidamente no tecido fibroso. **Manobra de Mendelsohn:** foi eficaz para eliminar a aspiração, promover contato completo da base da língua com a parede posterior da faringe e aumento de sua duração do contato (1 estudo), aumento da duração da elevação laríngea de forma consistente e melhora da duração do fechamento laríngeo. Além disso a manobra proporcionou melhora em 80% dos pacientes na ingestão oral em pelo menos 1 pontuação na escala do FOIS (1 estudo) **Supraglótica:** para o paciente com ressecção composta da área do trígono retromolar direito a manobra promoveu poucos benefícios em relação a alteração na base de língua, na abertura faríngea e esofágica superior durante a deglutição e na fisiologia da deglutição (1 estudo).	Atualmente, existem evidências limitadas de seis estudos que mostram os efeitos positivos das intervenções comportamentais de deglutição para populações com distúrbios estruturais. Por causa da gama de déficits estruturais resultantes do câncer e de seus tratamentos, são necessárias mais pesquisas que avaliem a eficácia da intervenção específica.
Park et al.^(33)^, 2021 Coreia J Oral Rehabil. (3.837)	Investigar os protocolos de exercícios, métodos e ferramentas usados ​​em vários estudos de exercícios CTAR e resumiu seus achados.	Embase, Medline e Cochrane library	**Intervenção:** 30 a 42 sessões, 30 min, 5 a 7 dias por semana e tratamento com duração de 4 a 8 semanas. **Controle**: NI	**CTAR vs exercício de shaker:** ambos promoveram melhoras significativas nas fases oral e faríngea (2 estudos) e nos escores de PAS. O CTAR também promoveu melhora na fisiologia da cavidade oral na deglutição, elevação laríngea e fechamento epiglótico, diminuição de resíduo valecular e resíduo nos seios piriformes. **Exercício CTAR:** promoveu significativamente maiores valores médios e picos de ativação do músculo supra-hióideo e causou menor ativação do músculo esternocleidomastóideo. **CTAR vs tratamento de disfagia tradicional:** CTAR apresentou maior melhora significativa na PAS do que o tratamento tradicional. **CTAR vs. Shaker vs. exercício de flexão do queixo com *Theraband:*** aumento significativo na pressão anterior da língua com CTAR e com a flexão do queixo com *Theraband.*	O exercício CTAR ativa mais seletivamente o músculo supra-hióideo e é um exercício terapêutico eficaz para melhorar a função de deglutição em pacientes com disfagia. Por ser menos extenuante do que o exercício de Shaker, requer menos carga física e esforço, permitindo uma maior adesão.
Schwarz et al.^(45)^, 2018 Austrália Int J Lang Commun Disord. (3.020)	Realizar uma revisão sistemática da eficácia da estimulação termo-tátil (ETT) como ferramenta compensatória e/ou reabilitadora.	CINAHL, Medline e SpeechBite	**Intervenção**: 8 a 20 sessões, 30 min (relatado em 1 estudo). **Controle**: NI	Diminuição do tempo de trânsito faríngeo; Redução mediana no tempo de trânsito oral; Diminuição da duração total da deglutição (p = 0,005); diminuição do tempo de trânsito total (diminuição de 69%, p = 0,049 para fluidos e de 77%, p = 0,033 para pastoso); Redução mediana no tempo de trânsito faríngeo para fluidos = 0,2 (redução de 85%, 0,004), pasta = 0,3 (redução de 85%, p = 0,011); Redução mediana no tempo de trânsito total para fluidos de 69%, p = 0,049 e para pastoso 77%, p = 0,033); Redução mediana no tempo de atraso faríngeo para fluidos de 92%, p = 0,002 e para pasta redução de 69%, p = 0,196; Melhora da resposta de latência da deglutição utilizando massagem com gelo do que sem massagem com gelo (p = 0,0366).	Há evidências de baixo nível para apoiar o uso de ETT. A melhor prática atual seria usar o ETT caso a caso, seguindo uma avaliação instrumental detalhada e a avaliação de sua eficácia para um indivíduo.
Speyer et al.^(49)^, 2010 Holanda Dysphagia (3.438)	Relatar os efeitos da terapia da deglutição aplicada por fonoaudiólogos.	PubMed e Embase	**Intervenção**: NI **Controle**: NI	59 foram incluídos e em geral, foram encontrados efeitos positivos da terapia estatisticamente significativos. Contudo, o número de estudos era pequeno. Além disso, diversos problemas metodológicos foram encontrados em muitos desses estudos.	A comparação foi dificultada pela variedade de diagnósticos, tipos de terapias e técnicas de avaliação. Embora alguns estudos de resultados positivos significativos tenham sido publicados, mais pesquisas baseadas em ensaios clínicos randomizados são necessárias.
Sun et al.^(38)^, 2020 China Am J Phys Med Rehabil. (2.159)	Avaliar a eficácia da estimulação elétrica neuromuscular transcutânea nos distúrbios da deglutição.	MEDLINE / PubMed, Embase, CENTRAL, Web of science e PEDro	**Intervenção**: 10 a 20 sessões, 16 a 60 min, 2 a 6 vezes por semana (maioria 5 vezes por semana). **Controle**: NI	Em comparação com os grupos de controle, EENM e TT melhorou significativamente a função de deglutição por um DMP de 0,62 (95% CI = 0,06 a 1,17; I2 = 89%). O DMP dos oito estudos restantes foi de 0,92 (95% CI = 0,19 a 1,64; I2 = 90%). **Grupos musculares de estimulação:** **Estudos estimulando grupos musculares supra-hióideos:** valor DMP negativo de 0,17 (IC 95% = −0,42 a 0,08) sem heterogeneidade significativa (I2 = 0%). **Estudos estimulando os grupos musculares infra-hióideos** (DMP = 0,89; 95% IC = 0,47 a 1,30; I2 = 0%) **Estudos estimulando os grupos musculares supra-hióideos e infra-hióideos** (DMP = 1,40; 95% IC = 1,07 a 1,74; I2 = 91%). **Reações adversas:** não foi relatado nenhum efeito adverso grave associado à EENM, houve apenas o relato de queixa de dor (2 estudos), dor transitória, que desapareceu imediatamente após a interrupção da EENM (1 estudo) e queixa de dor leve, que cessou após o ajuste da intensidade de estimulação (1 estudo).	Não há evidências firmes para concluir sobre a eficácia da estimulação elétrica neuromuscular nos distúrbios da deglutição. Ensaios clínicos controlados randomizados bem desenhados e em maior escala são necessários para se chegar a conclusões robustas.
Tan et al.^(39)^, 2013 China J Oral Rehabil. (3.837)	Avaliar a eficácia geral da estimulação elétrica neuromuscular transcutânea e da TT, comparando os dois protocolos de tratamento	PubMed/Medline, Cochrane Central Register of Controlled Trials e EMBASE MEDLINE	**Intervenção:** 10 a 20 sessões, 30 min a 1 hora, 5 vezes na semana. **Controle:** 13 a 18 sessões, 30 a 60 min, frequência média de 5 sessões semanais durante 3 semanas	Melhora significativa foi observada no grupo EENM em comparação com o grupo TT no DMP 0,77 (IC de 95%: 0,13 a 1,41, p = 0,02). A heterogeneidade tornou-se pequena (I^2^ = 0%). O EENM foi significativamente superior ao TT com um valor de resultado geral agrupado de 0,5 (95% IC: 0,2 a 0,8, p = 0,001). Parece que o resultado foi relativamente estável. Uma análise de sensibilidade adicional foi realizada excluindo o estudo em que os pacientes do GC terminaram o tratamento em casa. Com o valor global do resultado combinado de 0,46 (95% IC: 0,15 a 0,77, p =0,004), parece haver diferenças estatisticamente significativas entre os dois métodos. O resultado foi relativamente estável. Na análise de subgrupo de acordo com a etiologia da disfagia, não houve diferença significativa entre EENM e TT no grupo AVC, que teve um valor DMP agrupado de 0,78 (95% IC: −0,22 a 1,78, p = 0·13, 4 estudos, 175 pacientes). No entanto, a análise de subgrupo de pacientes sem AVC, incluindo câncer e pacientes com DP, revelou diferenças estatisticamente significativas entre as duas intervenções, e o valor DMP geral combinado foi de 0,63 (95% IC: 0,24 a 1,02, p = 0·002).	A meta-análise mostrou que a EENM é mais eficaz para o tratamento de pacientes adultos com disfagia de etiologias variáveis do que a TT. Mas, em pacientes com disfagia após acidente vascular cerebral, a eficácia de EENM e TT foi comparável. Considerando as limitações descritas acima, deve-se ter cautela ao interpretar nossos achados. Recomendamos que a EENM seja útil para o tratamento da disfagia, estudos de alta qualidade com grande número de pacientes são necessários.
Yang et al.^(35)^, 2015, Coréia do Sul Dysphagia (3.438)	Avaliar a eficácia e segurança da estimulação cerebral não invasiva em pacientes com disfagia após acidente vascular cerebral.	Medline, EMBASE e Biblioteca Cochrane	**Intervenção:** 5 a 10 sessões, 10 a 30 min, 5 a 7 vezes na semana. **Controle**: 5 a 10 sessões, 10 a 30 min, 5 a 7 vezes na semana.	Melhora estatisticamente significativa em pacientes com disfagia que foram tratados com NIBS imediatamente após a estimulação em comparação com os pacientes que foram submetidos a estimulação simulada (DMP = 1,08, IC de 95% = 0,29-1,88, I^2^ = 72%). Resultados das avaliações em 1 mês após a estimulação (DMP = 2,75, IC de 95% = 1,47-4,04, I^2^ = 70%) e 2 meses (DMP= 3,54, IC de 95% = 2,58 –4,50, I^2^ = 0%) revelou uma melhora estatisticamente significativa. Análise de subgrupos com base no uso de intervenção no grupo EMTr versus o grupo de estimulação simulada (DMP = 1,61, IC de 95% = 0,59–2,63, I^2^= 67%) revelou melhora significativa. Nenhuma diferença estatisticamente significativa no grupo ETCc versus o grupo de estimulação simulada (DMP = 0,54, IC de 95% = -0,05-1,62, I^2^= 68%). Na análise de subgrupos com base no local de estimulação, o grupo de estimulação do local contralesional apresentou melhora estatisticamente significativa em comparação com o grupo de estimulação simulada (DMP = 0,90, IC de 95% = 0,16-1,64, I^2^ = 0%), enquanto o grupo de estimulação do local ipsilesional não apresentou melhora (DMP = 1,015, IC de 95% = -0,69–2,79, I^2^ = 88%). Nenhuma diferença estatisticamente significativa entre o grupo de estimulação ipsilesional e o grupo de estimulação contralesional (I^2^= 0%, p = 0,87).	Os resultados indicam que o tratamento com NIBS para disfagia após AVC tem um efeito benéfico em comparação com a estimulação simulada. Além disso, o NIBS revela efeitos sinérgicos ao longo do tempo. Na análise de subgrupo, a estimulação EMTr ofereceu efeitos benéficos em comparação com a estimulação simulada. Nenhuma diferença significativa de acordo com o local de estimulação (estimulação ipsilesional ou contralesional) foi observada. Nenhuma complicação de NIBS foi relatada nesta análise. O pequeno número de estudos e a falta de acompanhamento em longo prazo são as principais limitações desta revisão. Estudos futuros se beneficiariam da padronização dos resultados e parâmetros de estimulação para diminuir a variabilidade e heterogeneidade dos resultados e os resultados de longo prazo.
**Deglutição e Fala**					
Blyth et al.^(44)^, 2015 Austrália Int J Speech Lang Pathol. (2.484)	Relatar a intervenção fonoaudiológica na fala e função de deglutição após glossectomia parcial.	MEDLINE, CINAHL, PubMed, EMBASE, Scopus, AMED, Web of knowledge, EBM reviews e speechBITE.	**Intervenção**: 12 sessões, 30 min, 3 vezes na semana. **Controle:** NI	Em todos os estudos a terapia incorporou múltiplos exercícios e compensações ao invés de uma técnica singular. Em relação ao momento da intervenção no pós-operatório, o início do tratamento variou de 9 dias a 9 anos após a cirurgia. Os estudos analisados neste artigo abordam a intervenção na fala, outros na deglutição, 4 artigos discutem a intervenção na disfagia e 4 na articulação.	Há poucas publicações a respeito da reabilitação fonoaudiológica após glossectomia parcial, apresentando lacunas de suas evidências científicas
Gadenz et al.^(31)^, 2015 Brasil Folia Phoniatr Logop. (0.849)	Revisar sistematicamente ensaios clínicos randomizados que avaliam os efeitos da estimulação magnética transcraniana repetitiva (EMTr) nos aspectos de reabilitação relacionados às funções de comunicação e deglutição.	PubMed, Clinical Trials, Cochrane Library e ASHA.	**Intervenção**: 5 a 15 sessões, 10 a 30 min por dia, 1 vez ao dia 7 vezes na semana. **Controle:** 5 a 15 sessões, 10 min a 30 min por dia, 1 vez ao dia 7 vezes na semana.	Foram analisados ​​9 estudos: 4 sobre afasia, 3 sobre disfagia, 1 sobre disartria na DP e 1 sobre déficits linguísticos na doença de Alzheimer. Todos os estudos de afasia usaram EMTr de baixa frequência para estimular a área homóloga de Broca. A EMTr de alta frequência foi aplicada sobre o córtex faringoesofágico do hemisfério esquerdo e / ou direito nos estudos de disfagia e sobre o córtex dorsolateral pré-frontal esquerdo nos estudos de Parkinson e Alzheimer. Dois estudos de afasia e todos os estudos de disfagia mostraram uma melhora significativa do distúrbio, em comparação com o grupo placebo. Os outros 2 estudos relacionados à afasia encontraram benefício restrito a subgrupos com caso grave ou lesão na porção anterior da área cortical da linguagem, respectivamente, enquanto o Alzheimer o estudo demonstrou efeitos positivos específicos para a compreensão auditiva. Não houve mudanças para a função vocal no estudo de Parkinson.	Os benefícios da técnica e sua aplicabilidade em distúrbios neurogênicos relacionados à comunicação e deglutição ainda são incertos. Portanto, outros ensaios clínicos randomizados são necessários para esclarecer o protocolo de estimulação ideal para cada distúrbio estudado e seus reais efeitos.
**Respiração**					
Kayamori e Bianchini^(32)^, 2015 Brasil Rev. CEFAC	Analisar a literatura científica quanto às propostas de TMO em adultos com distúrbios respiratórios do sono, assim como seus efeitos sobre sintomas e parâmetros fisiológicos desses distúrbios.	Lilacs, MEDLINE, Pubmed, Cochrane e Scielo	**Intervenção:** NI **Controle**: NI	Foi observado que os efeitos mais relevantes da terapia miofuncional orofacial isolada em adultos incluem: redução da sonolência diurna e do ronco, melhor qualidade do sono, diminuição parcial do IAH e aumento parcial da saturação mínima sanguínea. Os ensaios clínicos randomizados controlados e cegos são poucos e são importantes para atestar os efeitos da técnica com base em evidências e orientar as decisões terapêuticas considerando a avaliação e o diagnóstico e o fenótipo do paciente para um prognóstico preciso.	Seis estudos mostraram diminuição do IAH, cinco estudos mostraram melhora da saturação mínima SpO2, pontuação da escala de sonolência e do ronco. Apesar das diferenças metodológicas, itens dificultadores quanto às comparações dos resultados, os estudos levantados confirmam os efeitos positivos da TMO para pacientes com AOS.

Legenda: DP = Doença de Parkinson; EENM = Estimulação elétrica neuromuscular; d = tamanho do efeito - Teste de Cohen; RR = risco relativo; FOIS = Functional oral intake scale; DOSS = Dysphagia Outcome and Severity Scale; FEES = videoendoscopia da deglutição; NI = não informado; CTAR = chin tuck against resistance; PAS = penetration-aspiration scale; sEMG = eletromiografia de superfície; SMD = média padronizada, EMTr = estimulação magnética transcraniana repetitiva; ETCc = estimulação transcraniana por corrente contínua; AOS = apnéia obstrutiva do sono; IAH = índice de apneia e hipopneia; DMP = diferença média padronizada; TT = terapia tradicional; NIBS = Neuromodulação não invasiva

As RSs incluídas relataram algumas limitações nos estudos primários como a heterogeneidade nas avaliações^(29,36,43)^, nas intervenções^(29,37,38,43,48,51)^ e na população estudada^(38,40,43)^; riscos de viés de seleção dos estudos primários^(45)^; dados limitados^(30,34,46,50,53)^; amostra pequena^(35,39,41,42,47,52)^, escassez de ensaios clínicos randomizados controlados^(40-42,48)^ e cegos^(31,45,46)^; falhas metodológicas nos estudos incluídos^(37)^, utilização de instrumentos subjetivos na avaliação^(35,49)^; além da inclusão de estudos apenas no idioma inglês^(37,38)^ e com nível de evidência baixa^(39,44)^.

As intervenções encontradas nas RSs selecionadas foram várias (Tabela 3), todavia as mais frequentes foram os exercícios de deglutição/terapia convencional (60%), EENM (44%) e manobras protetoras/facilitadoras da deglutição (40%) todas relacionadas a estudos na área da deglutição.

**Tabela 3:** Intervenções utilizadas

**Autores**	**Exercícios de deglutição/ terapia convencional**	**Modificação da dieta**	**Exercício de Shaker**	**Estimulação tátil-térmica (ETT)**	**Estimulação elétrica neuromuscular (EENM)**
	**Exercícios laríngeos e faríngeos**	**Alimentação não oral (enteral)**			**Biofeedback eletromiográfico**
	**Exercícios de mandíbula**	**Estimulação termo-tátil, física e olfatória**	**Exercícios para reabilitação para disfunção de fala e/ou deglutição**		**Estimulação magnética transcraniana repetitiva (EMTr)**
	**Exercícios de língua**			**Tratamento de voz Lee Silverman Voice Treatment (LSVT)**	
	**Manobras protetoras/facilitadoras da deglutição**	**Treinamento de força muscular expiratória**	**Chin Tuck Against Resistance (CTAR)**		**Estimulação magnética transcraniana contínua**
	**Exercícios miofuncionais/motores orofaciais**				
Banda et al.^(36)^	X	X	X	X	X
Park et al.^(34)^	X X	X			X
Alamer et al.^(48)^	X				X
Albuquerque et al.^(29)^	X				X
Andrade et al.^(30)^	X X X			X	
Antunes et al.^(42)^	X X X		X		
Ashford et al.^(53)^	X				X
Battel et al.^(50)^	X				X
Benfield et al.^(46)^	X				X
Blyth et al.^(44)^			X		
Carnaby e Madhavan^(41)^	X X	X			X
Chen et al.^(37)^	X				X X
Cousins et al.^(47)^	X X X	X		X	X X
Dionísio et al.^(43)^					X
Foley et al.^(51)^		X X X			
Gadenz et al.^(31)^					X
Kayamori e Bianchini^(32)^	X				
López-Liria et al.^(52)^	X X X	X X		X	X X
McCabe et al.^(40)^	X				
Park et al.^(33)^	X X X	X	X		
Schwarz et al.^(45)^				X	
Speyer et al.^(49)^	X X X	X		X X	X
Sun et al.^(38)^					X
Tan et al.^(39)^	X				X
Yang et al.^(35)^					X X

## RESULTADOS INDIVIDUAIS DOS ESTUDOS

### Deglutição e fala

Foram encontrados dois estudos que avaliaram a deglutição e fala, sendo que um deles analisou pacientes pós glossectomia parcial, abordando exercícios para reabilitação da disfunção de fala/deglutição^(44)^ e outro avaliou a EMTr em uma população pós AVC, Parkinson ou Alzheimer^(31)^.

Nos dois estudos a análise dos dados foi qualitativa. Ainda há muitas lacunas a respeito da reabilitação fonoaudiológica na população estudada^(44)^, porém um dos estudos^(31)^ conseguiu concluir que há benefícios da técnica aplicada, todavia ainda são necessários mais estudos.

### Respiração

Foi encontrada apenas uma RS^(32)^ que analisou as propostas da terapia miofuncional em adultos com distúrbios respiratórios do sono. Os resultados foram apresentados de forma qualitativa, apontando efetividade na redução da apneia, melhora da saturação mínima e melhora na pontuação da escala de sonolência e do ronco.

### Deglutição

As RSs encontradas abordaram a intervenção na deglutição (para casos de disfagia e um estudo que incluiu idosos saudáveis) por meio de exercícios de deglutição e uso de dispositivos como estimulação elétrica neuromuscular^(36,48)^, terapia fonoaudiológica da deglutição^(30,34,49)^, biofeedback eletromiográfico e/ou terapia fonoaudiológica^(29)^, exercício de shaker^(42)^, manobras (queixo para baixo, inclinação lateral, rotação da cabeça, mendelsohn e manobra supraglótica)^(53)^ (queixo para baixo, deglutição com esforço, Mendelsohn, deglutição supraglótica, super-supraglótica)^(40)^, *biofeedback* eletromiográfico^(46,50)^, estimulação elétrica neuromuscular^(37)^, estimulação magnética transcraniana^(43)^, *Chin Tuck Against Resistance* (CTAR)^(33)^, estimulação termo-tátil^(45)^, estimulação elétrica neuromuscular transcutânea^(38,39)^, estimulação cerebral não invasiva (Estimulação transcraniana por corrente contínua (ETCC) e EMTr^(35)^. Em quatro^(41,47,51,52)^ estudos, os autores analisaram as opções existentes de intervenções na reabilitação da deglutição.

### Avaliação da qualidade metodológica e qualidade da evidência

Cinco RSs^(34,35,38,39,46)^ foram julgadas como tendo baixa confiança, enquanto as outras 20 RSs^(29-33,36,37,40-45,47-53)^ foram julgadas como tendo uma confiança criticamente baixa. Nenhuma das revisões cumpriu todos os requisitos das diretrizes do AMSTAR (Figura 2).

**Figura 2:** AMSTAR II: ferramenta de avaliação crítica para revisões sistemáticas

Todos as RSs analisadas cumpriram os pré requisitos dos domínios 3, 4, 10 e 16 do AMSTAR, sendo eles respectivamente, justificativas para a seleção do desenho do estudo (domínio 3), estratégias de busca (domínio 4), relato da fonte de financiamento para estudos incluídos (domínio 10) e relato do conflito de interesses dos autores da revisão (domínio 16). Apenas oito artigos cumpriram os pré-requisitos em relação aos domínios 9 e 13, correspondentes a técnica usada para avaliar o risco de viés dos estudos incluídos (domínios 9) e sobre consideração do risco de viés na interpretação e discussão dos resultados (domínios 13). Nenhum dos artigos cumpriu o domínio 2, que se refere ao relato do registro do protocolo a priori.

## DISCUSSÃO

Tendo em vista a necessidade que a literatura apresenta sobre as evidências dos tipos de intervenção para treinar as funções orofaciais em indivíduos sem disfunções, e para reabilitar as disfunções orofaciais, este estudo realizou um mapeamento das evidências existentes através de RSs disponíveis sobre as intervenções fonoaudiológicas nas áreas de respiração, mastigação, deglutição e fala. Vinte e cinco RSs que atenderam aos critérios de elegibilidade foram considerados para a análise.

As RSs incluídas foram publicadas entre 2008 e 2021, e dessas, 11 (44%) foram publicadas nos últimos cinco anos^(29,33,34,36,38,43,45,46,48,50,52)^. Em relação a idade, foi observado uma maior frequência de estudos que incluíram pacientes com mais de 50 anos (24%), oito RSs (32%) não informaram a idade e cinco RSs (20%) apresentaram apenas a média de idade. Essa variação na forma de apresentar as informações dificulta a inferência relacionada à idade.

Foram encontradas na literatura diversas intervenções abordando a deglutição (para casos de disfagia e um estudo que incluiu idosos saudáveis); dentre elas exercícios de deglutição, exercícios laríngeos e faríngeos, exercícios de mandíbula, exercícios de língua, manobras protetoras e facilitadoras da deglutição, exercícios miofuncionais/motores orofaciais, modificação da dieta, alimentação não oral (enteral), estimulação termo tátil, física e olfatória, treinamento de força muscular expiratória, exercício de Shaker, exercícios para reabilitação para disfunção de fala e/ou deglutição, CTAR, ETT, LSVT, além de tratamentos com auxílio dos dispositivos como a EENM, *biofeedback* eletromiográfico, EMTr. As intervenções abordando a deglutição e fala incluíram EMTr e exercícios para reabilitação. As intervenções abordando respiração incluíram terapia miofuncional. As intervenções mais utilizadas foram os exercícios de deglutição e/ou terapia convencional, seguindo de EENM e manobras protetoras e facilitadoras da deglutição, todas relacionadas a estudos na área de deglutição. Os exercícios de deglutição e manobras protetoras e facilitadoras são propostas com eficácia comprovada, descritas ao longo dos anos^(54)^. A EENM vem sendo citada como tendo um importante papel em vários segmentos na clínica da reabilitação da disfagia, mas autores descrevem a importância de especificar a etiologia da disfagia para comprovar o benefício na prática fonoaudiológica^(17)^.

Alguns estudos apresentaram conclusões com evidências positivas. Uma RS concluiu que os exercícios de deglutição foram eficazes na melhora da função de deglutição (imediatamente e após a intervenção) e na abertura da boca (imediatamente, após a intervenção e no acompanhamento de até seis meses)^(36)^. Mais cinco RSs também apresentaram conclusões com evidências científicas positivas de acordo com seus objetivos^(29,30,33,37,48)^.

Também tiveram RSs em que os autores descreveram os resultados, mas relataram em suas conclusões dados insuficientes^(31,34,44)^, resultados promissores, porém, falta de estudos mais robustos^(35,41,42,46,47,50,52)^, evidências limitadas^(38,40,43,49,51,53)^ e evidências de baixo nível^(45)^. Estes dados corroboram com uma revisão de literatura realizada em 2007^(55)^ onde a autora relata sobre as mesmas dificuldades e limitações encontradas nos trabalhos de eficácia da reabilitação em disfagia orofaríngea. Todavia, é importante destacar que o estudo de escopo não aborda o peso relativo da evidência em favor da eficácia das intervenções analisadas, mas sim um relato narrativo ou descritivo da pesquisa disponível^(56)^.

As RSs apresentam alto nível de evidências científicas, possuindo um bom desenho de estudo e metodologia rigorosa para embasar a PBE, fornecendo evidências com alta confiabilidade e com menor risco de viés^(57)^, contudo elas estão vulneráveis a alguns tipos de vieses, por isso a interpretação dos resultados deve ser feita com cautela. Nesta revisão de escopo as RSs mostraram limitações específicas aos estudos secundários, algumas limitações como a heterogeneidade entre os estudos^(29,37,48,51)^, pequenos tamanhos de amostra^(35,39,41,42)^, além de apontarem a necessidade de mais estudos com alta qualidade metodológica^(40,42,47,51-53)^.

Nos domínios^(2,9,15)^ do AMSTAR 2 todos os artigos cumpriram os pré-requisitos. No domínio 7, 20 artigos (80%) cumpriram e no domínio 5, 16 artigos (64%). Em contrapartida, no domínio 1, apenas 9 artigos atingiram os pré-requisitos, no domínio 6, apenas 12 artigos cumpriram os pré-requisitos.

Dos domínios considerados críticos^(2,3,6,8,12,14)^ pela ferramenta AMSTAR 2 foi observado que todos os artigos cumpriram os pré-requisitos do 4, já no 2 nenhum dos artigos apresentavam uma declaração clara de que os métodos de revisão foram definidos antes da revisão. Em relação ao 7, apenas 4 artigos (16%) cumpriram parcialmente os pré-requisitos e 21 artigos (84%) atenderam a todos os requisitos. Os domínios 9 e 13, 17 artigos (68%) não cumpriram os pré-requisitos. Em relação ao domínio 15 apenas 6 (24%) realizaram síntese quantitativa, na qual 5 (20%) artigos realizaram uma investigação adequada do viés de publicação e discussão em seus resultados. Nenhuma das revisões cumpriu todos os requisitos das diretrizes do AMSTAR 2, demonstrando a necessidade de estudos com melhor rigor metodológico.

### Implicações para a pesquisa

A revisão de escopo realizada permitiu mapear as sínteses de evidências sobre as intervenções fonoaudiológicas nas áreas de deglutição, fala e respiração, em adultos e idosos. Não foi encontrada RS sobre a função de mastigação. Foi possível observar diversos tipos de intervenções na área da deglutição, desde a terapia tradicional até a utilização de dispositivos.

Contudo, devido às limitações dos estudos, os dados devem ser interpretados com cautela. Também são necessárias pesquisas com alta qualidade metodológica a respeito das intervenções fonoaudiológicas para inferências a respeito das evidências com maior confiança, podendo assim, selecionar RSs com estudos mais robustos. Há limitações nessa revisão de escopo quanto à qualidade metodológica e de evidência das RSs incluídas, além da ausência de RSs com foco específico na análise de segurança.

## CONCLUSÃO

A revisão de escopo realizada permitiu mapear as sínteses de evidências sobre as intervenções fonoaudiológicas nas áreas de deglutição, fala e respiração, em adultos e idosos. Foi possível observar diversos tipos de intervenções na área da deglutição, da terapia tradicional até a utilização de dispositivos. Contudo, devido às limitações dos estudos, os dados devem ser interpretados com cautela. Também são necessárias pesquisas com alta qualidade metodológica a respeito das intervenções fonoaudiológicas para inferências e generalizações com maior confiança a respeito das evidências de eficácia, e principalmente da segurança.

## Apêndice A Estratégia de Busca

**Table:**

Database	Pesquisa (9 de agosto de 2021)
Cochrane	(“therapeutics” OR “therapy” OR “Therapeutic” OR “Therapies” OR “Treatment” OR “Treatments” OR “rehabilitation” OR “intervention” OR “interventions”) in Title Abstract Keyword AND (“mastication” OR “Chewing” OR “Deglutition” OR “Deglutitions“ OR “Swallowing“ OR “Swallowings” OR “Speech” OR “Respiration” OR “Breathing”) in Title Abstract Keyword AND (“systematic review” OR “systematic reviews as topic” OR “meta-analysis” OR “meta-analysis as topic” OR “network meta-analysis” OR “network meta-analysis”) in Title Abstract Keyword
3	
Embase	('therapeutics':ab,ti OR 'therapy':ab,ti OR 'therapeutic':ab,ti OR 'therapies':ab,ti OR 'treatment':ab,ti OR 'treatments':ab,ti OR 'rehabilitation':ab,ti OR 'intervention':ab,ti OR 'interventions':ab,ti) AND ('mastication':ab,ti OR 'chewing':ab,ti OR 'deglutition':ab,ti OR deglutitions:ab,ti OR swallowing:ab,ti OR 'swallowings':ab,ti OR 'speech':ab,ti OR 'respiration':ab,ti OR 'breathing':ab,ti) AND ('systematic review':ab,ti OR 'systematic reviews as topic':ab,ti OR 'meta-analysis':ab,ti OR 'meta-analysis as topic':ab,ti OR 'network meta-analysis':ab,ti)
LILACS	(“therapeutics” OR “therapy” OR “Therapeutic” OR “Therapies” OR “Treatment” OR “Treatments” OR “rehabilitation” OR “intervention” OR “interventions” OR “Terapéutica” OR “Terapêutica” OR “Terapia” OR “Terapias” OR “Tratamento” OR “Tratamentos” OR “Speech Therapies” OR “Fonoterapia” OR “Logopedia”) AND (“mastication” OR “masticación” OR “mastigação” OR “mastication” OR “chewing” OR “Deglutição” OR “Deglutition” OR “deglutitions“ OR “swallowing“ OR “swallowings” OR “Deglución” OR “Speech” OR “Fala” OR “Habla” OR “Respiration” OR “Breathing” OR “Respiração” OR “Respiración”) AND (“systematic review” OR “Revisão Sistemática” OR “Revisión Sistemática” OR “meta-analysis” OR “meta-analysis as topic” OR “metanálise” OR “Metaanálisis” OR “network meta-analysis” OR “network meta-analysis” OR “Metanálise em Rede” OR “Metaanálisis en Red”) AND (db:(“LILACS”))
192	
LIVIVO	TI=((“therapeutics” OR “therapy” OR “Therapeutic” OR “Therapies” OR “Treatment” OR “Treatments” OR “rehabilitation” OR “intervention” OR “interventions”)) AND TI=((“mastication” OR “Chewing” OR “Deglutition” OR “Deglutitions“ OR “Swallowing“ OR “Swallowings” OR “Speech” OR “Respiration” OR “Breathing”)) AND TI=((“systematic review” OR “systematic reviews as topic” OR “meta-analysis” OR “meta-analysis as topic” OR “network meta-analysis” OR “network meta-analysis”))
3104	
PubMed/ Medline	(“therapeutics”[MesH] OR “therapeutics” OR “therapy” OR “Therapeutic” OR “Therapies” OR “Treatment” OR “Treatments” OR “rehabilitation”[MesH] OR “intervention” OR “interventions”) AND (“mastication”[MesH] OR “Chewing” OR “Deglutition”[Mesh] OR “Deglutitions“ OR “Swallowing“ OR “Swallowings” OR “Speech”[Mesh] OR “Respiration”[Mesh] OR “Breathing”) AND (“systematic review”[Publication Type] OR “systematic reviews as topic”[MesH] OR “systematic review” OR “meta-analysis” OR “meta-analysis as topic”[MesH] OR “meta-analysis” OR “network meta-analysis”[MesH] OR “network meta-analysis”)
1642	
Web of Science	(“therapeutics” OR “therapy” OR “Therapeutic” OR “Therapies” OR “Treatment” OR “Treatments” OR “rehabilitation” OR “intervention” OR “interventions”) (Topic) AND (“mastication” OR “Chewing” OR “Deglutition” OR “Deglutitions“ OR “Swallowing“ OR “Swallowings” OR “Speech” OR “Respiration” OR “Breathing”) (Topic) AND (“systematic review” OR “systematic reviews as topic” OR “meta-analysis” OR “meta-analysis as topic” OR “network meta-analysis” OR “network meta-analysis”) (Topic)
2253	
Scopus	TITLE-ABS (“therapeutics” OR “therapy” OR “Therapeutic” OR “Therapies” OR “Treatment” OR “Treatments” OR “rehabilitation” OR “intervention” OR “interventions”) AND TITLE-ABS (“mastication” OR “Chewing” OR “Deglutition” OR “Deglutitions“ OR “Swallowing“ OR “Swallowings” OR “Speech” OR “Respiration” OR “Breathing”) AND TITLE-ABS (“systematic review” OR “systematic reviews as topic” OR “meta-analysis” OR “meta-analysis as topic” OR “network meta-analysis” OR “network meta-analysis”)
ASHA Wire	(“therapeutics” OR “therapy” OR “Therapeutic” OR “Therapies” OR “Treatment” OR “Treatments” OR “rehabilitation” OR “intervention” OR “interventions”) AND (“mastication” OR “Chewing” OR “Deglutition” OR “Deglutitions“ OR “Swallowing“ OR “Swallowings” OR “Speech” OR “Respiration” OR “Breathing”) AND (“systematic review” OR “systematic reviews as topic” OR “meta-analysis” OR “meta-analysis as topic” OR “network meta-analysis” OR “network meta-analysis”)
1271	
Google Scholar	“therapeutics” AND “mastication” OR “Deglutition” OR “Speech” OR “Respiration” AND “systematic review” OR “meta-analysis”
17400	
Open Grey	“therapeutics” OR “intervention” AND “systematic review”
623	
Proquest	(“therapeutics” OR “therapy” OR “Therapeutic” OR “Therapies” OR “Treatment” OR “Treatments” OR “rehabilitation” OR “intervention” OR “interventions”) AND (“mastication” OR “Chewing” OR “Deglutition” OR “Deglutitions“ OR “Swallowing“ OR “Swallowings” OR “Speech” OR “Respiration” OR “Breathing”) AND (“systematic review” OR “systematic reviews as topic” OR “meta-analysis” OR “meta-analysis as topic” OR “network meta-analysis” OR “network meta-analysis”)
66184	
SpeechByte	“therapeutics” OR “intervention” AND “mastication” OR “Deglutition” OR “Speech” OR “Respiration” AND “systematic review” OR “meta-analysis”
	“ Treatments” AND “mastication” OR “Deglutition” OR “Speech” OR “Respiration” AND “systematic review” OR “meta-analysis”
	“Mastication” OR “Deglutition” OR “Speech” OR “Respiration” AND “systematic review” OR “meta-analysis”
	“rehabilitation” AND “mastication” OR “Deglutition” OR “Speech” OR “Respiration” AND “systematic review” OR “meta-analysis”
	“Treatments” OR “rehabilitation” OR “intervention” OR “interventions”) AND (“mastication” OR “Chewing” OR “Deglutition” OR “Deglutitions“ OR “Swallowing“ OR “Swallowings” OR “Speech” OR “Respiration” OR “Breathing”) AND (“systematic review” OR “systematic reviews as topic” OR “meta-analysis” OR “meta-analysis as topic” OR “network meta-analysis” OR “network meta-analysis”)
